# β-carbonic anhydrases play a role in salicylic acid perception in Arabidopsis

**DOI:** 10.1371/journal.pone.0181820

**Published:** 2017-07-28

**Authors:** Laura Medina-Puche, María José Castelló, Juan Vicente Canet, Julián Lamilla, María Laura Colombo, Pablo Tornero

**Affiliations:** Instituto de Biología Molecular y Celular de Plantas (IBMCP); Universitat Politècnica de València (UPV)-Consejo Superior de Investigaciones Científicas (CSIC); Valencia, Spain; Indiana University, UNITED STATES

## Abstract

The plant hormone salicylic acid (SA) is required for defense responses. *N**ON EXPRESSER OF* *P**ATHOGENESIS* *R**ELATED* *1* (*NPR1*) and *N**ON* *R**ECOGNITION OF* *B**TH-**4* (*NRB4*) are required for the response to SA in Arabidopsis (*Arabidopsis thaliana*). Here, we isolated several interactors of NRB4 using yeast two-hybrid assays. Two of these interactors, βCA1 and βCA2, are β-carbonic anhydrase family proteins. Since double mutant *βca1 βca2* plants did not show any obvious phenotype, we investigated other βCAs and found that NRB4 also interacts with βCA3 and βCA4. Moreover, several βCAs interacted with NPR1 in yeast, including one that interacted in a SA-dependent manner. This interaction was abolished in loss-of-function alleles of NPR1. Interactions between βCAs and both NRB4 and NPR1 were also detected *in planta*, with evidence for a triple interaction, NRB4-βCA1-NPR1. The quintuple mutant *βca1 βca2 βca3 βca4 βca6* showed partial insensitivity to SA. These findings suggest that one of the functions of carbonic anhydrases is to modulate the perception of SA in plants.

## Introduction

Salicylic acid (SA) is a plant hormone that regulates several aspects of plant development [1], although this hormone has primarily been studied in the context of biotic stress responses [2].

From early on, there has been much interest in finding the receptor (or receptors) for SA. Thus, several biochemical searches for SA binding proteins (SABP) were conducted in *Nicotiana tabacum* L., revealing the catalase SABP1, the methyl salicylate esterase SABP2, and the chloroplastic carbonic anhydrase SABP3 ([3][4][5]] respectively). Dozens of other proteins have since been defined as SABPs in Arabidopsis (*Arabidopsis thaliana*) using refined approaches [6]. *NPR1* was identified in several genetic screens involving the analysis of more than 50 Arabidopsis *npr1* mutant alleles that do not respond to SA [7][8][9][10][11]. NPR1 has been proposed to be the receptor for SA [12], although there are other candidates as well (see below). NPR1 is a protein with a BTB/POZ (broad-complex, tramtrack, and bric-à-brac/poxvirus and zinc-finger) domain, an ankyrin repeat domain, and a nuclear localization sequence. NPR1 regulates transcription by binding to transcription factors and is itself subjected to regulation via altered localization, degradation, and monomerization [2]. Investigations of NPR1 have led to the identification of other proteins involved in SA perception, such as suppressors, interactors, and paralogs of *NPR1* ([13][14][15] respectively). The TGA family of transcription factors interacts with NPR1 and with the promoters of pathogenesis-related proteins [16]. Members of the TGA family are also required for SA signaling, and a triple *TGA* mutant has an intermediate SA perception phenotype [17]. NIMIN1 [18] is a protein that interacts with NPR1 and negatively regulates its function. NPR3 and NPR4, that are NPR1 paralogs, bind to SA and were proposed to be SA receptors and to regulate the degradation of NPR1 [19].

Mutations in *NRB4* also render plants unresponsive to SA [20]. NRB4 is a likely homolog of MED15, a subunit of the Mediator complex. This 22-protein complex functions as a bridge between specific transcription factors that interact with *cis* elements of a promoter and RNA Pol II [21]. NRB4 consists of a small (approximately 100 amino-acids [aa]) KIX conserved domain [22], where point mutations are located, and 1200 remaining aa with a Gln-rich region in the middle [20]. Plants with point mutations in *NRB4* have no response to SA, whereas knockout mutants are sterile, with severely affected growth as well as insensitivity to SA [20]. As previously mentioned, SABP3 is a carbonic anhydrase (CA, EC 4.2.1.1). CA enzymes interconvert water and CO_2_ into HCO_3_^-^, an activity essential for all organisms [23]. Mutations in *CA* genes reduce the response of the plant to different CO_2_ levels [24][25], as well as the response to pathogens [26]. *CAs* are highly conserved through the tree of life and include three families in plants, α, β, and γ [27]. SABP3 was first described in tobacco as a chloroplast protein that binds SA [5], and belongs to the β family, which has six members in Arabidopsis (TAIR10, www.arabidopsis.org).

In this study, we found that several βCAs interact with SA, NRB4 and NPR1, bringing together two proteins defined in genetic screens of Arabidopsis as necessary for SA perception. A quintuple mutant lacking all but one member of the βCAs family is compromised in its response to SA, demonstrating that βCAs are relevant in SA perception.

## Materials and methods

### Plant growth and inoculation

Arabidopsis (*Arabidopsis thaliana* [L.] Heynh.) and *Nicotiana benthamiana* were grown as described [28], in controlled environment rooms with 8 h days at 21°C, 150 μmol m^-2^ s^-1^ of light intensity, and 16 h nights at 19°C. There was no CO_2_ regulation. The treatments, inoculations, and sampling began 30 minutes after the beginning of artificial day to ensure reproducibility. The following genotypes were used: *npr1*-1 [29], *npr1*-70 [11], *nrb4*-1 and *nrb4*-2 [20], *NahG* [30], *eds5* [31], and *sid2* [32]. The T-DNA insertion lines used were βCA1, SALK_106570; βCA2, GK-036A01; βCA3, SALK_032009; βCA4, WiscDsLox508D11; βCA5, WiscDsLoxHs003_12H, SALK_009250, and DsLoxHs105_09G; and βCA6, SALK_044658. The *βca1 βca4 βca6* line was constructed by [24].

*Pseudomonas syringae pv*. *tomato* DC3000 (*Pto*) was grown, inoculated, and measured as described by [33]. Briefly, 14-day-old plants were inoculated by spraying with *Pto* at OD_600_ 0.1 with 0.02% Silwet L-77 (Crompton Europe Ltd., Evesham, UK). Three days later, the amount of colony forming units (cfu) per plant was quantified and represented on a logarithmic scale. Other strains used were *Pto(avrRpm1)* [34], *Pto(avrRpt2)* [35], *Pto(avrPphB)* [36], *Pto(avrRps4)* [37], and *Pto(hopZ1a)* [38]. In the eds-like experiment, 12 seven-week-old plants were hand inoculated with a needleless syringae containing *Pto* at OD_600_ 10^−4^. Three leaves per plant were completely infiltrated and three days later, the inoculated leaves were collected, weighed, and the amount of bacteria measured. For all experiments, at least three independent treatments were performed (three independent sets of plants sown and treated on different dates). Statistical analyses were performed with Excel 2007 (Microsoft, Redmond, WA, USA) and R [39].

### Chemical treatments

Primers and chemical products were purchased from SIGMA (St. Louis, MO, USA) unless otherwise stated. Benzothiadiazole (BTH, CGA 245704), in the form of commercial product (Bion^®^ 50 WG, a gift from Syngenta Agro S.A., Madrid, Spain), was prepared in water for each treatment and applied with a household sprayer. The response to BTH (in terms of fresh weight) was measured as reported [28]. Briefly, plants were treated with mock or 350 μM BTH four times over a three-week period. The fresh weight of the plants were then recorded and expressed as the ratio between BTH- and mock-treated plants. SA (in the form of sodium salicylate) was applied at 1 mM unless otherwise stated.

### SA in plates and *in planta*

Arabidopsis seeds were surface-sterilized for 10 min in ethanol and for 10 min in 1% formaldehyde, followed by five washes with distilled water. The seeds were plated on medium containing 0.5x Murashige and Skoog salts (Duchefa BV, Haarlem, the Netherlands), 0.6% (w/v) Phyto Agar (Duchefa), 2% (w/v) sucrose, and 0, 200, or 300 μM SA (final concentration). The results were evaluated 7 days after transfer to growth conditions. Chlorophyll was extracted from the plants with ethanol for 2 hours at 65°C and quantified as described by [40]. Three replicates of 10 plants each per treatment and genotype were measured. For *in planta* SA measurements, three approximately 100 mg samples of two weeks old plants were frozen in liquid nitrogen, and SA extraction was performed as described by [41] and [42].

### Yeast experiments

βCAs cDNAs were cloned in pDONR222 (Invitrogen, Barcelona, Spain) for RT-PCR and transferred to pDEST22 and pDEST32 (Invitrogen) for expression in yeast. Additionally, the pARC352 vector [43] was used for the triple interaction assay. For the initial Y2H screening, a custom cDNA library was produced with RNA pooled from Arabidopsis treated with SA and BTH at different times using a CloneMiner II kit (Invitrogen) in pDEST22. The primers used are listed in S1 Table. Yeast n-hybrid analyses were performed as described [44]. Briefly, yeast was transformed with two cDNAs, one in pDEST22 and one in pDEST32. Yeast growth on a plate lacking histidine was considered to indicate an interaction. βCA5.1, βCA6.2, and βCA6.5 were successfully cloned in pDEST32, but no yeast transformants were recovered using these clones, regardless of the presence of pDEST22. The interactions were quantified based on β-galactosidase activity, as described by [45]. In short, liquid cultures of each genotype were grown and their OD_600_ recorded. Then, 1.5 mL of sample for each data point was resuspended in Z-buffer, ortho-nitrophenyl-β-galactoside was added to initiate the reaction, and the time was recorded. Once a yellow color developed, the reaction is stopped by adding Na_2_CO_3_, and the time (in minutes) and OD_420_ were recorded. The activity, expressed as Miller units, is defined as: (OD_420_*1000)/(OD_600_*time*0.75 mL). When indicated, the yeast plates or liquid media were supplemented with 100 μM SA, BTH, 3-hydroxybenzoic acid (3HBA), 4-hydroxybenzoic acid (4HBA), acetazolamide (AA), ethoxyzolamide (EZ), or sulfanilamide (SU).

### Expression *in planta* and microscopy

The *βCA* cDNAs were transferred to pMDC43 ([46]; *GFP-βCAs*) and pB7FWG2 ([47]; *βCAs-GFP*) for expression *in planta*. For the BiFC experiments, the βCAs, NPR1s, and NRB4 cDNAs were cloned in pYFC43 and pNFC43 [48]. For the triple hybrid experiment, the plasmid pGWB15 [49] was used, and to investigate the interaction *in planta*, the plasmid pMDC-MBP was also used (N-terminal fusions of Maltose Binding Protein, a gift from Drs. Carrasco and Vera, IBMCP). *N*. *benthamiana* leaf tissue was mounted in water under a coverslip 4 days after infiltration with *Agrobacterium tumefaciens* containing the appropriate constructs. When indicated, plants were sprayed with 1 mM SA and collected or visualized one day later. The transgenic Arabidopsis plants were three weeks old when photographed. A Leica TCS SL confocal laser scanning microscope (Leica, Heidelberg, Germany) with an HCX PL APO CS 40X/1.25 water objective was used to study the subcellular localization of the fluorescence-tagged proteins. Green fluorescent protein was visualized by 488-nm excitation with an Ar laser, and its emission was examined with a 500 to 530 nm band-pass filter.

### Immunoblot and co-sedimentation assays

Crude protein extracts were prepared by homogenizing ground frozen leaf material in Tris-buffered saline (TBS) supplemented with 50 mM DTT and protease inhibitor cocktail. Protein concentration was measured using Bradford reagent, and 25 μg of total protein was separated by SDS-PAGE (10% acrylamide w/v) and transferred to a nitrocellulose blotting membrane. The membrane was stained with Ponceau-S after transfer for use as a loading control.

For co-sedimentation assays, extracts were cleared by two successive centrifugation steps at 16,000 x g and 4°C for 30 minutes, followed by incubation at 4°C for 1 h with amylose resin (New England Biolabs, Schwalbach, Germany) with gentle rocking. Unbound proteins were removed by successively washing the resin with TBS, TBS 0.5% (v/v) Triton X-100, and TBS 0.3 M NaCl, 1% (v/v) NP-40. Sedimented proteins were eluted with Laemmli sample buffer by heating for 5 minutes at 95°C. For immunoblot analysis, the following antibodies were used: rabbit polyclonal anti-GFP N-terminal antibody (G1544, used at 0.5 μg/mL, Sigma-Aldrich) and rabbit polyclonal anti-maltose binding protein antibody (ab 9084, used at 1:2000, Abcam, Cambridge, UK). The secondary antibody was anti-rabbit IgG-peroxidase conjugate (A6154, used at 1:2500, Sigma-Aldrich), and Amersham ECL Plus Western Blotting detection reagents (GE HealthCare, Little Chalfont, UK) and an LA-3000 Luminescent Image Analyzer (Fujifilm Life Science, Stamford, CT, USA) were employed.

### CA activity

Total CA activity was determined as described by [50]. The CA assay reaction mixtures contained a 50-μL aliquot of homogenate (yeast lysate or plant tissue homogenate) and 200 μl of pre-chilled 20 mM Tris-HCl buffer, pH 8.3, with 0.2% bromothymol blue. The assay was initiated by the addition of 700 μL of cold CO_2_-saturated water, and the time it took for the color to change from blue to yellow (pH 8.3 to 5.5) was recorded. Units of enzyme activity were calculated as: (T_b_/-T_s_)/(T_s_*P), where T_b_ and T_s_ represent the time (in sec) it took for the color to change in the blank and sample reaction, respectively, and P represents the amount of protein tested (in μg). Plant tissue of two weeks of age was homogenized in 0.2 M Tris-HCl buffer, pH 8.3, 1 mM EDTA, 20 mM MgCl_2_, 50 mM NaCl, and 0.1 M Na_2_SO_4_ at a proportion of 3:1 (volume: weight). Yeast was homogenized in the same buffer with 0.1% Triton X-100, and 1 mL of saturated culture was resuspended in the buffer.

### Protein expression in *E*. *coli*

NPR1 and βCA cDNAs were cloned into pHMGWA and expressed as described [51]. The optimal temperature was empirically determined for each fusion protein. The cells were harvested by centrifugation and the pellet resuspended into lysis buffer (20 mM Tris pH 7.4, 200 mM NaCl, 1 mM EDTA, 1 mM DTT, 0.2 mM PMSF, and protease inhibitor cocktail). Resuspended cells were disrupted by sonication, and cell debris was removed by centrifugation. An aliquot of clarified supernatant was loaded onto an SDS-PAGE (12% acrylamide w/v) gel to verify protein expression induction by Coomassie staining. Each clarified lysate was purified by amylose affinity chromatography (New England Biolabs) at 4°C and washed with 10 bed volumes of column buffer (20 mM Tris-HCl, pH 7.4, 200 mM NaCl, 1 mM EDTA, 1 mM DTT, 0.2 mM PMSF). Proteins that were bound to the amylose resin were eluted in column buffer supplemented with 10 mM maltose. Fractions containing purified protein were collected and stored at −80°C until use.

### SA-binding activity

As described in [52], purified proteins were incubated in the dark for 1 h on ice with 50 μM 4-AzSA (Santa Cruz Biotechnology, Dallas, TX, USA) in column buffer, followed by UV irradiation with 254 nm UV light at an energy level of 50 mJ, using a Stratalinker UV crosslinker 1800 chamber (Stratagene, Cedar Creek, TX, USA). The reaction mixtures were subjected to SDS-PAGE, and 4-AzSA crosslinked proteins were detected by immunoblot analyses using a sheep anti-SA antibody (20–1594, used at 1:1000, Fitzgerald Industries International, Acton, MA, USA). As a control for protein input, an aliquot of each reaction mixture was immunodecorated with rabbit polyclonal anti-MBP antibody described above. The secondary antibody was anti-sheep IgG-peroxidase conjugate (A3415, used at 1:2500, Sigma-Aldrich),

### Accession numbers

Sequence data from this article can be found in the Arabidopsis Genome Initiative or GenBank/EMBL databases under the following accession numbers: NRB4, At1g15780; NPR1, At1g64280; βCA1, AT3G01500; βCA2, AT5G14740; βCA3, AT1G23730; βCA4, AT1G70410; βCA5, AT4G33580; βCA6, AT1G58180.

## Results

### NRB4 interacts with CAs

We previously characterized *NRB4* [20] as a likely ortholog of *MED15* in Arabidopsis. MED15 is a member of the Mediator complex in eukaryotes [53]. Point mutations in the conserved KIX domain, or knockout insertions in *NRB4*, produce plants that are unable to perceive SA [20]. To further characterize the function of NRB4 in the perception of SA, we searched for proteins that could interact with NRB4 in the yeast two-hybrid system (Y2H). We identified 70 genes, among which only one, *βCA1*, also known as *SALICYLIC ACID BINDING PROTEIN 3* (*SABP3*, [26]), was proposed to function in defense or SA signaling. Interestingly, we also identified another member of the same *CA* family, *βCA2*, among the genes that interacted with NRB4 in Y2H.

**Fig 1:**
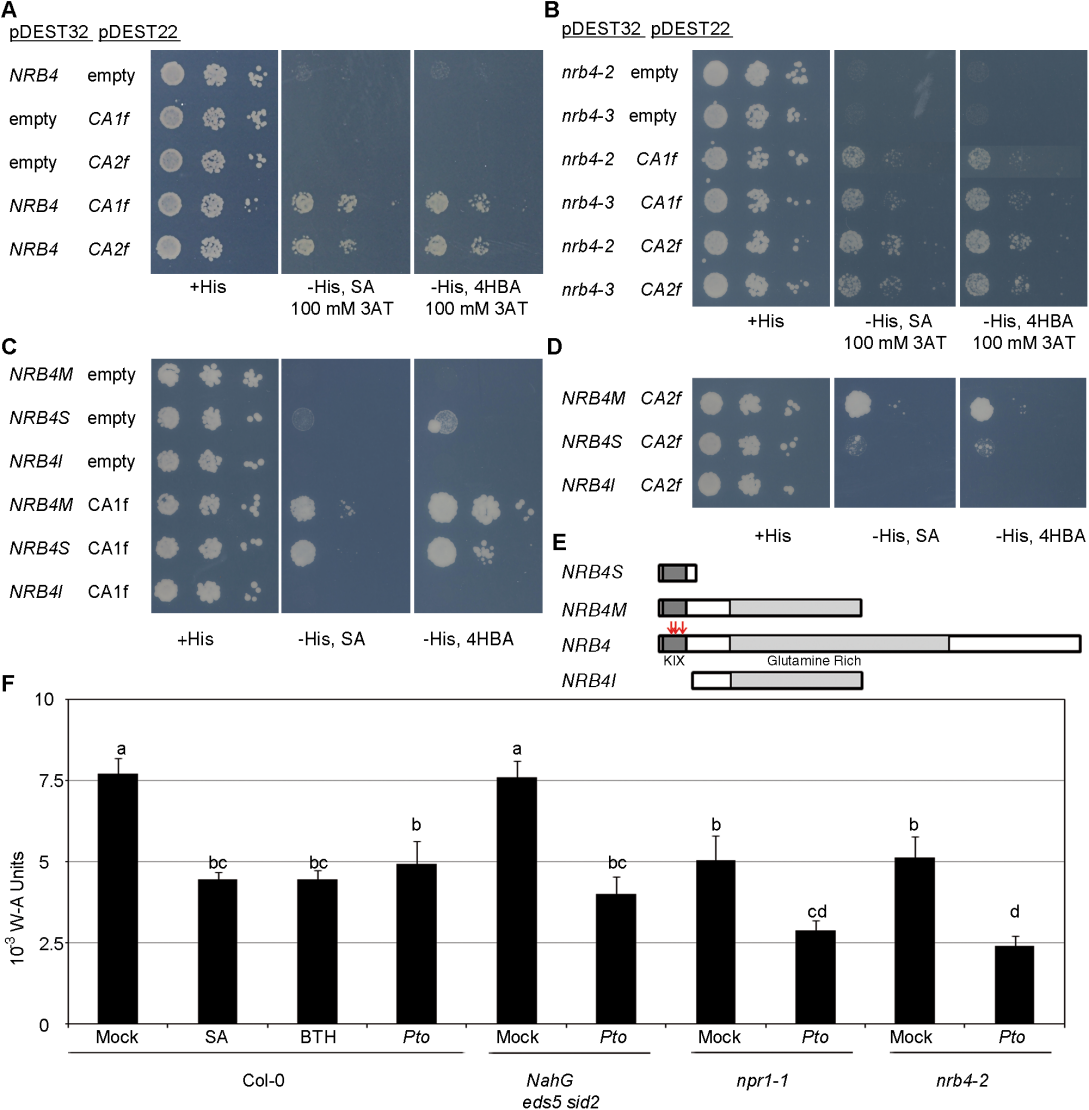
NRB4 interacts with βCA1f and βCA2f. (A) Yeast cells transformed with the indicated plasmids and inserts were grown on three different sets of plates. The first set contained minimal medium supplemented with histidine (+His), the second contained the same minimal medium with no histidine (-His), 100 μM salicylic acid (SA), and 100 mM 3-Amino-1,2,4-triazole (3AT), and the third lacked histidine (-His) and contained 100 μM 4-hydroxybenzoic acid (4HBA) and 100 mM 3AT. The first three rows indicate that NRB4, βCA1f, or βCA2f alone do not allow the yeast to grow in medium lacking histidine. The growth of yeast in the remaining two rows on -His plates indicates that NRB4 interacts with βCA1f and βCA2f. Note that the presence of SA or 4HBA does not affect the interaction. (B) βCA1f and βCA2f interact with the point mutations of NRB4 *in planta*. The interactions between βCA1 and βCA2 with nrb4-2 and nrb4-3 were tested as mentioned in “A”. (C) βCA1f interacts with the KIX domain of NRB4. The interaction between βCA1f and three different constructs of NRB4 was tested as mentioned in “A”, except that the -His plates did not contain 3AT. (D) βCA2f interacts with the KIX domain of NRB4. The interaction between βCA2f and three different constructs of NRB4 was tested as mentioned in “C”, with the controls shown in “C”. (E) Diagram of the NRB4 constructs used in the previous panels. The names of the domains are under the wild type NRB4. The red arrows indicate the point mutations found [20]. (F) CA activity in different genotypes and under different treatments. Two weeks old Col-0 plants were treated with mock solution, 1 mM SA, or 350 μM benzothiadiazole (BTH), and the samples were frozen one day later. Similarly, Col-0, *NahG eds5 sid2*, *npr1*-1, and *nrb4*-2 plants were inoculated with *Pseudomonas syringae* pv. *tomato* isolate DC3000 (*Pto*) at an OD_600_ of 0.1, and the samples were frozen one day later. The samples (three repeats of approximately 100 mg each) were ground and CA activity measured based on the change in color of bromothymol blue following the change in pH. In all figures where numerical information is presented, the data represent the average values, with error bars showing standard deviation. The letters above the bars indicate different homogeneous groups with statistically significant differences (Fisher’s LSD Test, P < 0.05). All experiments were repeated at least three times with similar results.

In the Y2H analysis, NRB4 bound to fragments of βCA1 and βCA2 (denoted βCA1f and βCA2f, respectively) regardless of the presence of SA in the medium (Fig 1A). As a control, we used 4-hydroxybenzoic acid (4-HBA), an isomer of SA with no biological activity. To test the specificity of the binding, two of NRB4 mutations that make the plant unable to perceive SA were reproduced in Y2H. Both mutants forms, nrb4-2 and nrb4-3, bound to βCA1f and βCA2f (Fig 1B). To identify the region of NRB4 that binds to the CAs, we used several versions of NRB4 (Fig 1E). NRB4M, comprising aa 1 to 670, bound to both βCA1f (Fig 1C) and βCA2f (Fig 1D). NRB4S, comprising aa 1 to 112, bound βCA1f to some extent (Fig 1C), but only bound βCA2f poorly if at all. A third version, NRB4I, comprising aa 100 to 670, did not bind to any of the interactors (Fig 1C, 1D and 1E). Note that *NRB4M* complements *nrb4-2* and *nrb4-3* in stable transgenic plants, while *NRB4S* does not [20].

### Cloning and structure of βCAs in Arabidopsis

To investigate the relevance of CA activity in the *nrb4* mutants and in SA perception, we measured total CA activity in Arabidopsis plants. Plants treated with SA showed a marked reduction in CA activity. A similar reduction was found in plants treated with benzothiadizole (BTH, a functional analog of SA, [54]) or inoculated with the phytopathogen bacterium *Pseudomonas syringae* pathovar *tomato* isolate DC3000 (*Pto*) (Fig 1F, reported by [26]). The triple *NahG eds5 sid2*, a transgenic line with very low levels of SA [30] in a background with low SA biosynthesis [31][32], also showed low CA activity in response to inoculation with *Pto*, clearly indicating that there are effects by *Pto* independently of SA. Interestingly, basal CA activity was also reduced in *nrb4* and another mutant lacking SA perception, *npr1*, compared with the wild type. An additional reduction (compared to mock treatment) was observed when *nrb4* and *npr1* mutant plants were inoculated with *Pto* (Fig 1F). Treatment with SA decreased wild-type CA activity (Fig 1F) and in turn, decreasing the CA activity via treatment with the inhibitor ethoxyzolamide reduced the levels of SA *in planta* (S1A Fig). Together, all of these data suggest a connection between SA and CA activity.

**Fig 2:**
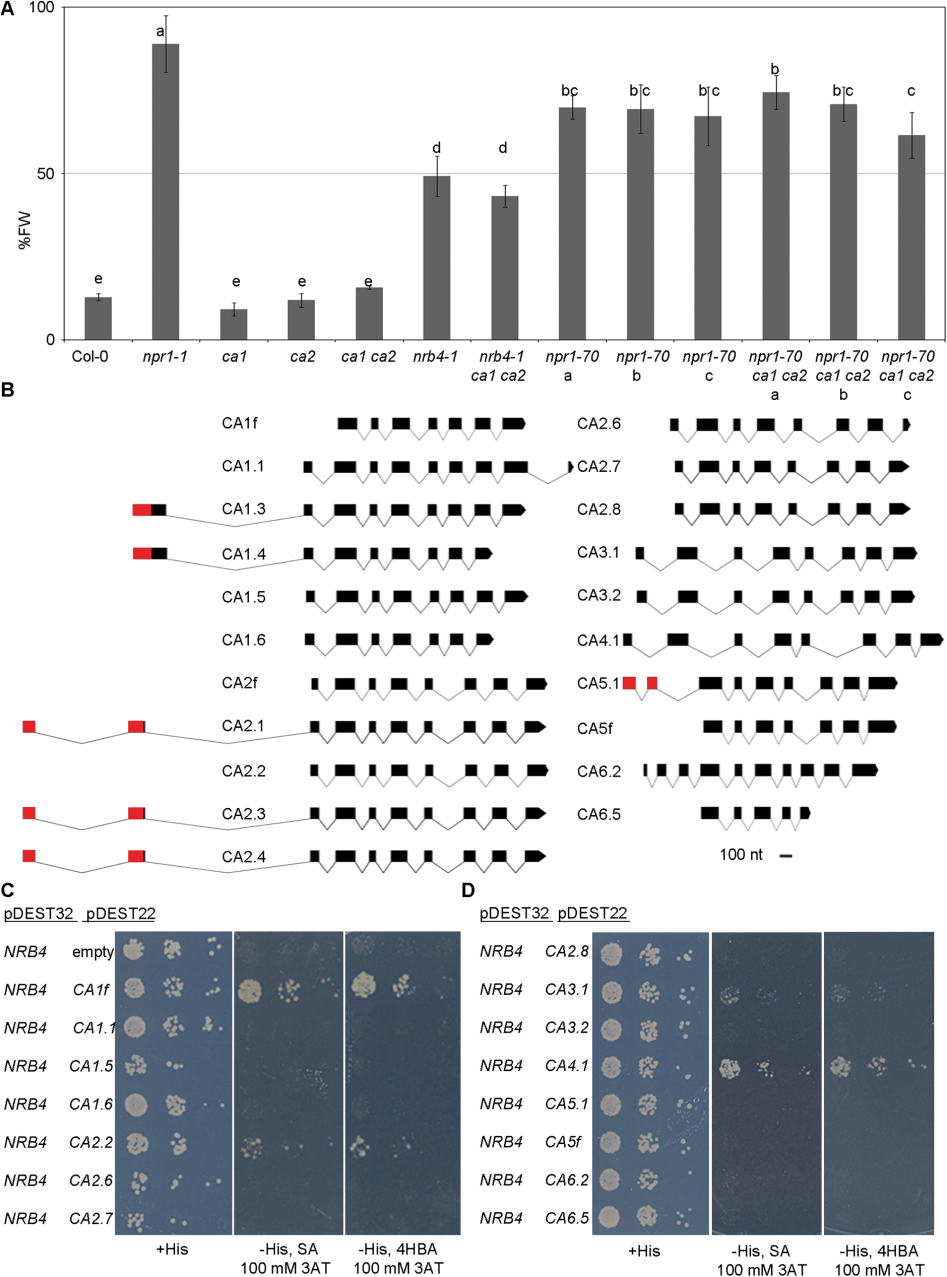
NRB4 also interacts with other βCAs. (A) Plants with T-DNA insertions in βCA1 (*βca1*) and βCA2 (*βca2*) were tested for their response to benzothiadizole (BTH, a functional analog of SA), along with some control genotypes, double, and triple mutants. The response to BTH was measured based on weight, and plants were treated with either mock solution or 350 μM BTH four times over the course of three weeks, their weights recorded, and the ratio between the BTH and mock-treated plants calculated (15 plants in three groups of five). The ratio is expressed as percentage of fresh weight (%FW). (B) Structures of the βCAs mentioned in this work. The black rectangles show the coding sequence of each cDNA. The red rectangles show the predicted chloroplastic peptide (www.cbs.dtu.dk/services/ChloroP/). The βCAs were manually aligned with the beginning of the third exon of βCA1.1 as a reference. (C) Six additional βCAs were tested as in Fig 1A. (D) Eight additional βCAs were tested as in Fig 1A. In total, βCA1f, βCA2.2, βCA3.1, and βCA4.1 interacted with NRB4.

The growth of plants lacking SA perception is unaffected by treatment with BTH [28], a phenotype that cannot be done with SA due to its phytotoxicity [55]. BTH treatment did have an effect on the growth of plants harboring T-DNA insertional mutations of *βCA1* and *βCA2* alone or in combination (Fig 2A). We further obtained triple mutants of these alleles with weak alleles of *npr1* or *nrb4* to test for subtle effects of the T-DNAs insertions in *βCA1* and *βCA2*, but observed none. Since the *βCA* gene family has six members, we considered the possibility that other genes besides *βCA1* and *βCA2* also function in SA perception. Broadly speaking, *βCA1*, *βCA2*, and *βCA4* were repressed upon inoculation with *Pto*, whereas *βCA6* was induced (S2A Fig). *βCA1* and *βCA2* had the highest mRNA levels in the *βCA* family, whereas *βCA3* had the lowest (S2B Fig). The *βCAs* had very little change in expression in response to BTH and in the *npr1* and *nrb4* backgrounds (S2C, S2D and S2E Fig). To investigate the hypothesis that additional genes in this family are involved in SA perception, we attempted to clone cDNAs of the *βCA* family members. This task was more difficult than expected, due to alternative splicing. We failed to identify some cDNAs described in the databases (TAIR10, www.arabidopsis.org) and found others not previously described (Fig 2B and S3 Fig, we only shown the ones found in this work). Since there are both chloroplastic and cytosolic versions of βCA1 and βCA2, we considered that there might be cytosolic forms of all chloroplastic βCAs found, thus creating βCA1.5, βCA1.6, βCA2.7, and βCA2.8 by using the same ATG that is used in the cytosolic βCA1.1 or in βCA2.2 (S3 Fig). In the case of *βCA5*, predicted to be chloroplastic, we produced a likely cytosolic version, βCA5f, starting with the first available ATG, as with *βCA1* and *βCA2*. We investigated the interactions between NRB4 and all of the βCAs (Fig 2C and 2D), finding that βCA1f, βCA2.2, βCA3.1, and βCA4.1 interacted with NRB4, with SA or 4HBA in the media. βCA1f is a fragment of βCA1.5, yet it behaves quite differently from this cDNA, apparently due to the lack of the first 24 aa (S3 Fig). For the experiments in *E*. *coli* or yeast, we did not use the cDNAs that produced *bona fide* chloroplastic proteins.

### NPR1 interacts with βCAs: Genetic specificity

**Fig 3:**
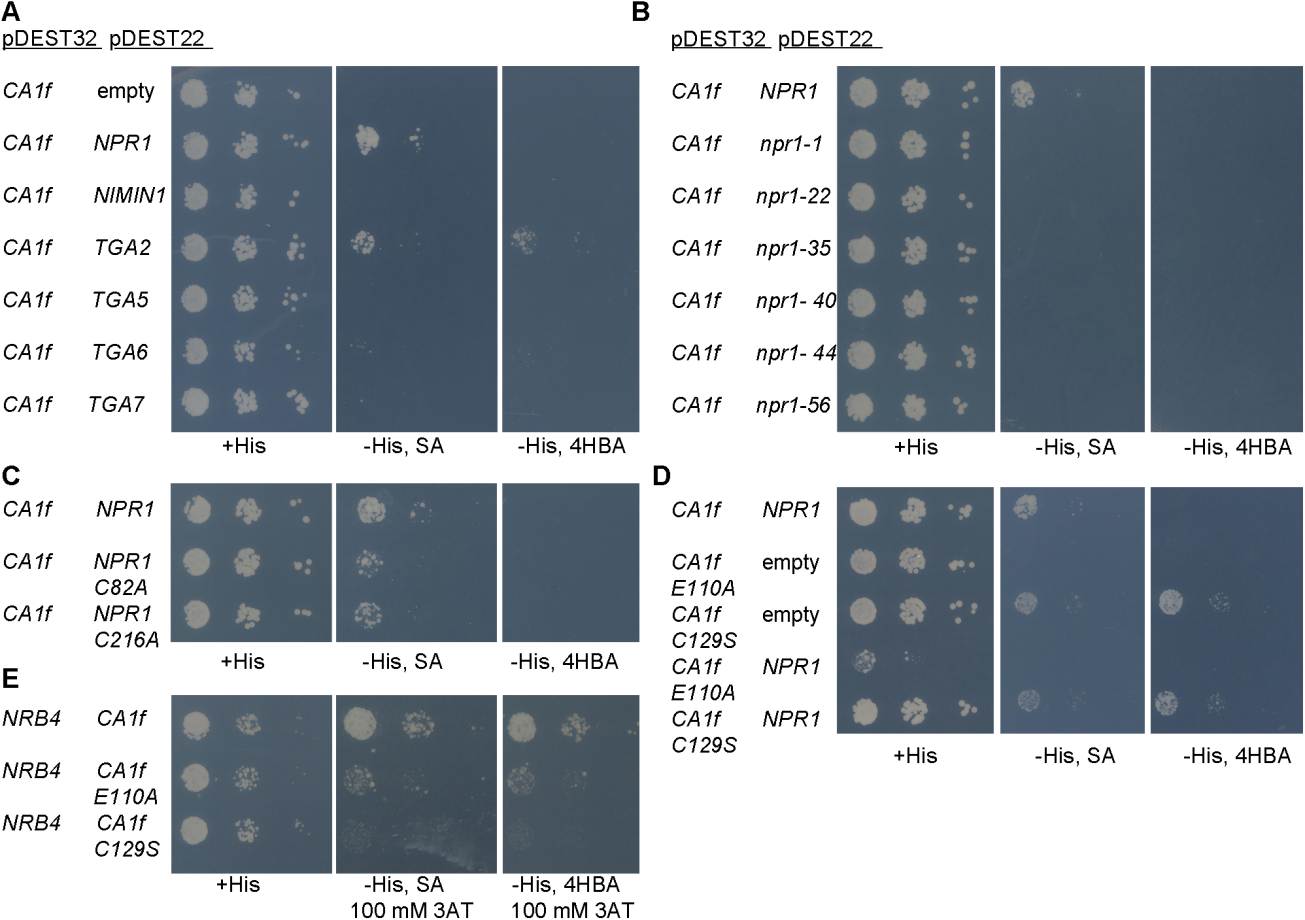
βCA1f interacts with NPR1 in the presence of SA. (A) The interactions between βCA1f and several proteins related to SA perception were tested as in Fig 1C. βCA1f interacts with NPR1 in a SA-dependent manner, while it interacts with TGA2 regardless of SA. (B) βCA1f did not interact with any of the six *npr1* alleles tested. These alleles are point mutations of NPR1 found *in planta*, and they produce stable protein. (C) βCA1f interacted with two NPR1 point mutations that do not alter NPR1 function [56]. (D) Mutations that disrupt CA activity affect the interaction between βCA1f and NPR1. Two mutations that produce stable pea CA with no activity [58] were re-created in βCA1f. (E) The same two mutations in βCA1f also affected the interaction between βCA1f and NRB4.

Since βCA1f was the strongest βCA interactor of NRB4, we examined the possibility that it also interacts with other proteins required for SA perception. Strikingly, βCA1f interacted with NPR1 in a SA-dependent fashion (Fig 3A). βCA1f also interacted with TGA2, regardless of the presence of SA, but it did not interact with NIMIN1, TGA5, TGA6, or TGA7 (Fig 3A). Numerous *npr1* alleles have been characterized *in planta* based on their lack of response to SA, and at least six randomly chosen alleles produce a stable protein [56]. However, none of these mutated versions of NPR1 interacted with βCA1f (Fig 3B), suggesting that any change in NPR1 protein might disrupt the interaction with βCA1f. We therefore tested two versions of NPR1, which had been constructed to investigate the behavior of NPR1 [57]. Although these proteins have a radical cysteine-to-alanine point mutation, they function as wild-type proteins, and, as shown in Fig 3C, they interacted with βCA1f.

Since there are no known mutations of βCA1 that affect its ability to bind SA, we focused on its CA activity itself. [58] found several point mutations in pea *CA* that produce a stable and soluble protein, but with no CA activity. We chose two of these mutations, E204A and C223S, and generated the equivalent mutations in βCA1f, E110A and C129S, respectively (S4A Fig). These mutations impaired the interaction between NPR1 and βCA1f, although βCA1f C129S exhibited some autoactivation (Fig 3D, control in S4B Fig). The same mutations in βCA1f also affected the interaction with NRB4, but βCA1f E110A was still able to interact weakly with this protein (Fig 3E).

### NPR1 interacts with βCAs: Chemical specificity

**Fig 4:**
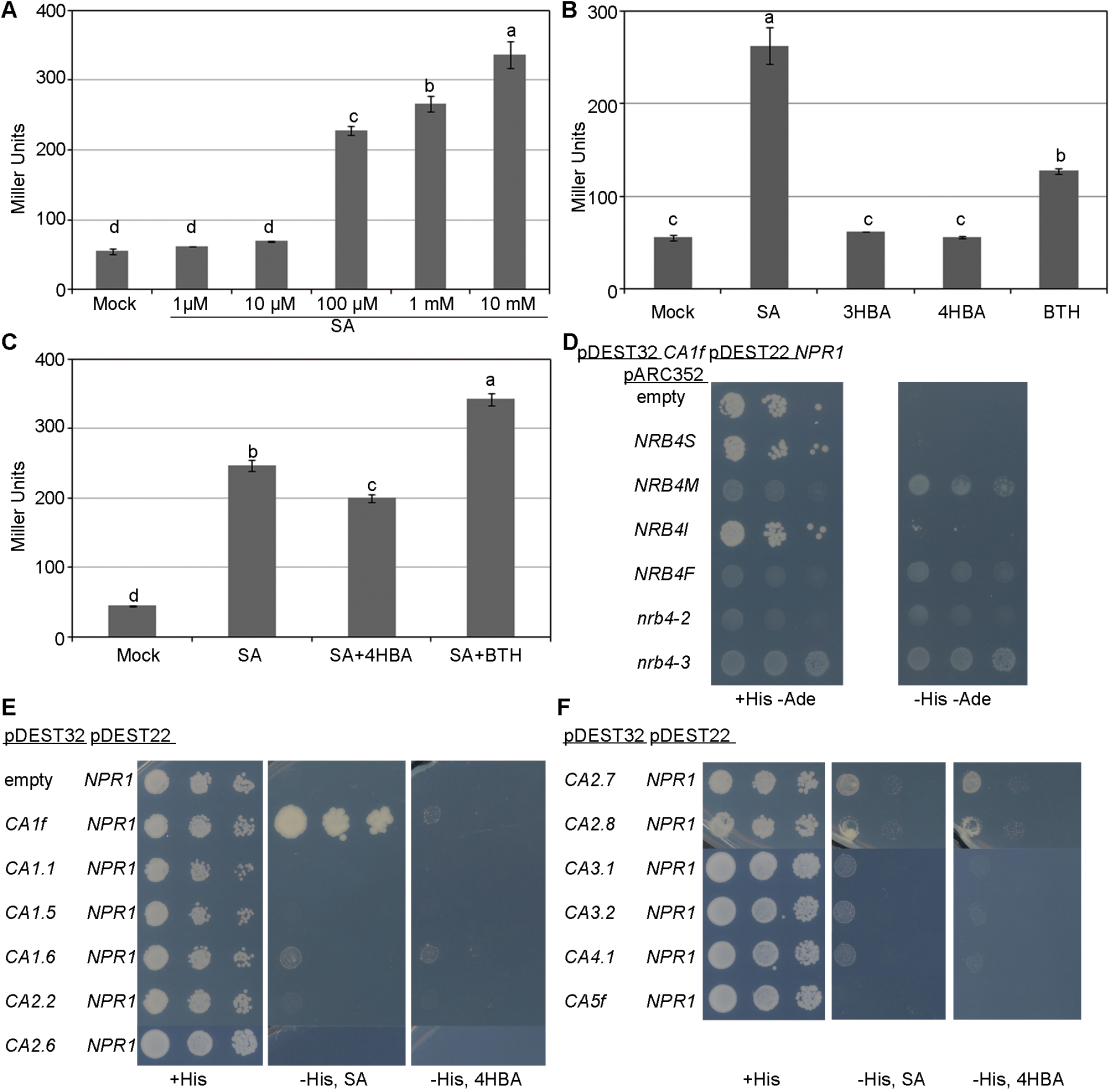
Quantification of the βCA1f-NPR1 interaction; other βCAs also interact with NPR1. (A) Dose response to SA in the interaction between βCA1f and NPR1. Yeast containing both cDNAs was grown in the presence of SA, and β-galactosidase activity was then measured, since the interaction between the two cDNAs leads to the expression of this enzyme. (B) The βCA1f-NPR1 interaction requires a functional analog of SA; 3HBA represents 3-hydroxybenzoic acid, and the interaction was quantified as described in “A”. (C) The inactive analogs competed poorly with SA, while active analogs showed an additive effect. The interaction was quantified as described in “A”. In panels “B” and “C”, 100 μM of each chemical was added to the medium. (D) Yeast three hybrid. Several versions of NRB4, described in Fig 1, were cloned in a third plasmid and introduced into yeast with βCA1f and NPR1 to show a triple interaction. (E) Five additional βCAs were tested as in Fig 1C. (F) Six additional βCAs were tested as in Fig 1C. In total, βCA1.6, βCA2.2, βCA2.7, βCA2.8, βCA3.1, βCA3.2, and βCA4.1 also interacted with NPR1, although less strongly and not depending on SA, as is the case with βCA1f.

Our above data indicated that interaction between NPR1 and βCA1f was dependent of SA. To address whether the concentrations of SA used in this study were non-physiological, or perhaps the interaction occurred only in a small range of SA concentrations, we quantified the NPR1-βCA1f interaction based on the production of the enzymatic product of the β-galactosidase [45]. The interaction was significant in the presence of at least 100 μM SA in the medium (Fig 4A), whereas chemical analogs of SA that do not trigger resistance in plants, i.e., 4HBA and 3-hydroxybenzoic acid (3HBA), did not increase the interaction between βCA1f and NPR1 (Fig 4B). Oddly, BTH, a strong inducer of resistance in plants [54], increased the interaction only to a certain degree. When 4HBA was added to the medium along with SA, there had a small negative effect on NPR1-βCA1f interaction (compared to SA alone). 4HBA likely competed for SA binding to CA1F and NPR1, reducing the SA-dependent interaction between the two proteins. When BTH was combined with SA, there was a notable additive effect, which is additional evidence for the specific effect of BTH on NPR1-βCA1f interaction (Fig 4C). In additional control experiments, SA and its analogs had no effects the growth or basal β-galactosidase activity of wild-type yeast or yeast containing NPR1 alone (S5A and S5B Fig, respectively), which indicates that there were no other targets in the assay besides the NPR1-βCA1f interaction. The CA activity inhibitors alone did not affect the interaction and reduced it only slightly when combined with SA (S5C Fig). Therefore, the reduction of CA activity caused by SA (Fig 1F) is not responsible for the NPR1-βCA1f interaction.

We previously found that NPR1 and NRB4 do not interact in Y2H [20]. Our current results showed that βCA1f interacted with both proteins in Y2H, suggesting that it might interact with both at the same time, perhaps functioning like a molecular bridge or scaffolding. As shown in S5D Fig, we did not detect such an interaction when βCA1f was introduced using a third plasmid. However, when NRB4 was introduced via a third plasmid, the interaction between βCA1f and NPR1 was altered and no longer required SA (Fig 4D). These results suggest that, instead of βCA1f working as a bridge between NPR1 and NRB4, NRB4 interacts with βCA1f, and this interaction facilitates the NPR1-βCA1f interaction. The same NRB4 constructs that interacted with βCA1f and βCA2f in Y2H (Fig 1A, 1B, 1C and 1D) produced this effect, and they also had a negative effect on the growth of yeast (Fig 4D). While we did not see a negative effect of NRB4 with N-terminal fusion in yeast, the negative effect of NRB4 with no fusions in yeast resembles the phenotype of NRB4 transgenic plants. In these plants, we could not recover a line in which NRB4 was detected [20], suggesting that NRB4 has deleterious effects in several organisms.

As with NRB4, we tested all available cDNAs from βCAs and identified more βCAs that interacted with NPR1. None of these interacted as strongly or as dependent on SA as βCA1f in the Y2H system, but βCA1.6, βCA2.2, βCA2.7, βCA2.8, βCA3.1, βCA3.2, and βCA4.1 interacted with NPR1 to various degrees (Fig 4E and 4F; note that βCA5.1, βCA6.2, and βCA6.5 could not be successfully transformed into yeast).

### Interactions *in planta*

**Fig 5:**
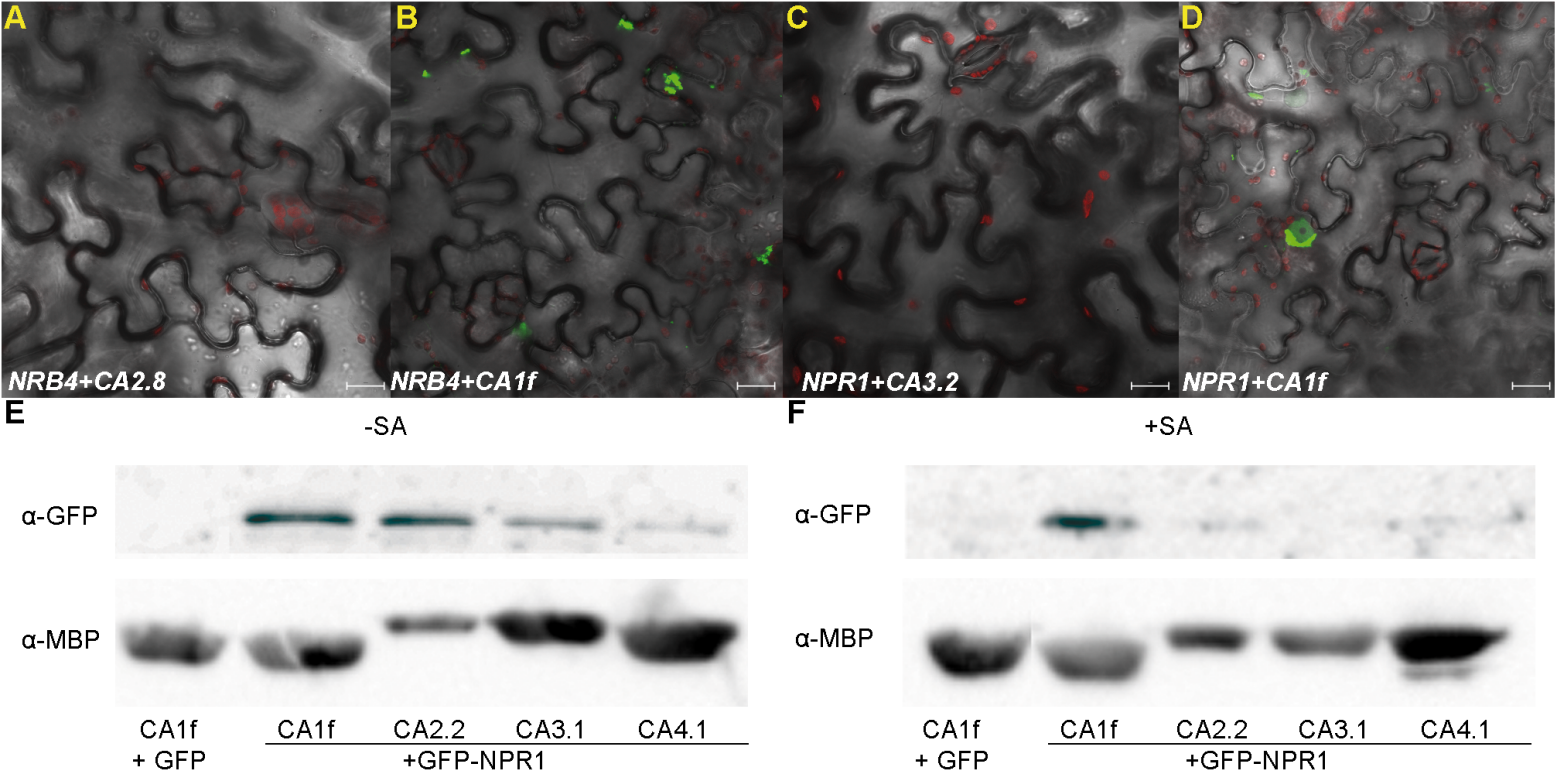
βCAs interact *in planta* with NRB4 and NPR1. Interaction of NRB4 with βCAs. (A) Bimolecular fluorescence complementation (BiFC) showing a negative interaction (as a control), and (B) positive interaction (detectable GFP). In total, four βCAs interacted with NRB4. Interaction of NPR1 with βCAs. Similarly, (C) shows a lack of interaction, while (D) shows a positive interaction. In total, six βCAs interacted with NPR1. The complete series of images can be found in S7 Fig. The bars represent 20 μm. (E) *GFP*-*NPR1* and different *MBP-βCAs* were transiently expressed in *N*. *benthamiana* by agroinfiltration and pulled-down with amylose resin. The panel shows the eluted fraction from the resin, detecting GFP-NPR1 (upper) and MBP-βCAs (lower) by immunoblot analysis with the indicated antibodies. (F) The same experiment after 1 mM SA treatment. Additional controls are showed in S9 Fig.

The interactions identified by Y2H were corroborated *in planta*. We monitored the SA levels produced in *Nicotiana benthamiana* upon inoculation with *Agrobacterium* alone or with constructs, since variation in SA levels could alter the results of the interaction assays. In two out of four experiments, *Agrobacterium* produced an increase in SA levels, while in the other two, there was no difference (S6 Fig). Therefore, we tested the interactions by Bimolecular Fluorescence Complementation (BiFC, [59]) in *N*. *benthamiana* leaves in the presence of exogenous SA to ensure reproducibility. NRB4 interacted with βCA1f, βCA2.2, βCA3.1, and βCA4.1, while NPR1 interacted with βCA1f, βCA2.2, βCA3.1, βCA4.1, βCA5.1, and βCA6.2 (Fig 5 and S7 Fig). Consistent with previous reports of NPR1 [57] and NRB4 [20] localization, the signals were localized to the nucleus (NPR1) and near the nucleus (NPR1 and NRB4; see magnified views in S8A, BS8, S8C and S8D Fig). As in yeast, we attempted to detect the triple interaction, NPR1-βCA1f-NRB4, *in planta*. NPR1 and NRB4 did not interact in the presence of a third empty vector, but when βCA1f was present in the third vector, a weak signal was observed (S8E, S8F, S8G, S8H and S8I Fig).

To further confirm these interactions, we co-expressed GFP-NPR1 and MBP-βCAs under mock conditions or after treatment with SA (Fig 5E and 5F). When the βCAs were pulled-down by affinity precipitation, NPR1 was detected in the precipitate, with no difference between mock and SA treatment for βCA1f and βCA4.1. In the case of βCA2.2 and βCA3.1, there is less NPR1 with SA (additional controls are shown in S9 Fig).

### Phenotypes of βCA T-DNA insertion mutants *in planta*

**Fig 6:**
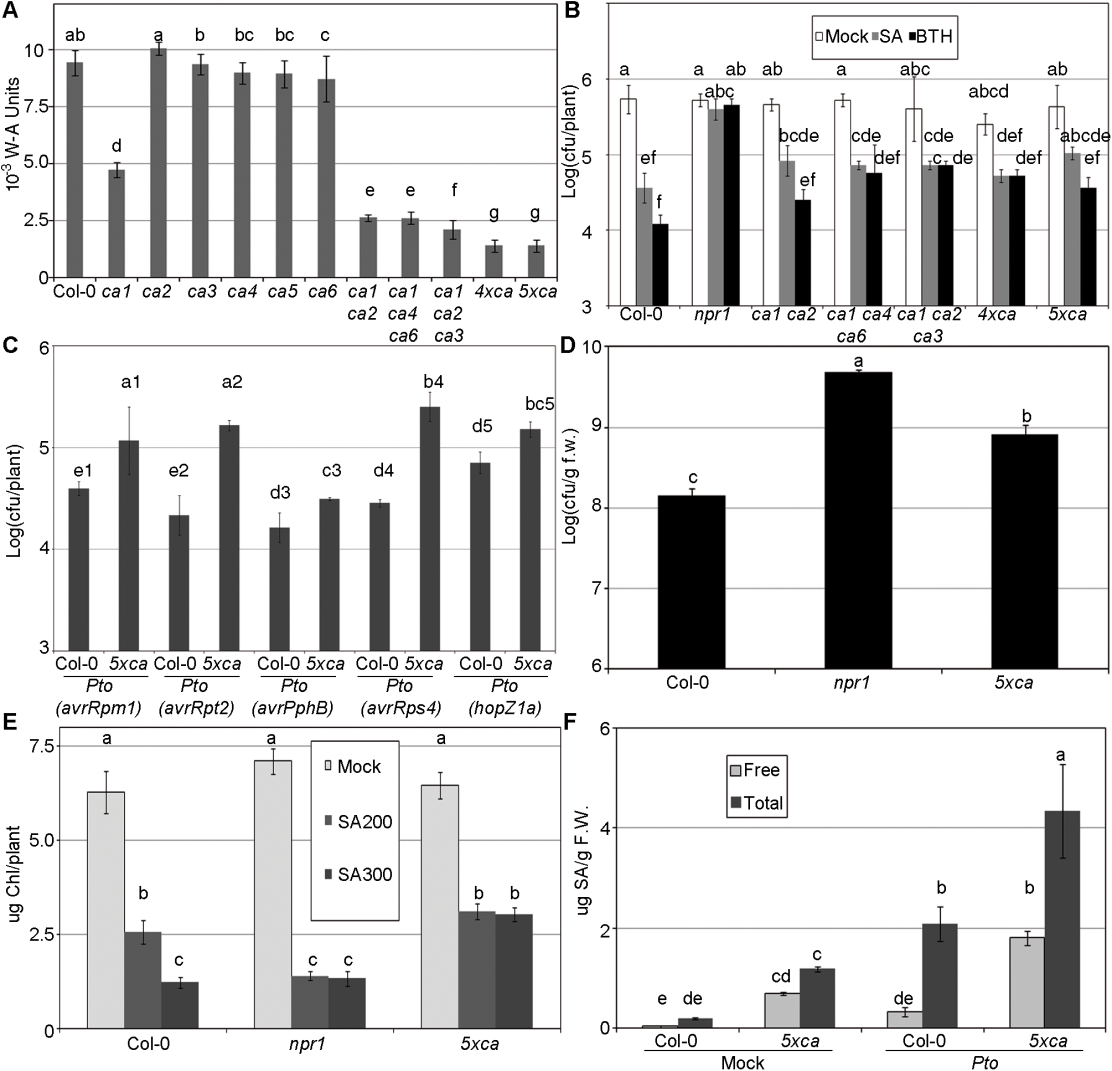
SA perception phenotypes of the βCA T-DNAs. (A) CA activity in plants with a single T-DNA insertion in the βCA genes and combinations of these mutants. In the case of *βca5*, the homozygous plant was sterile (S11 Fig), and the progeny of a heterozygous plant were used. The activity was measured as in Fig 1F; *4xβca* represents *βca1 βca2 βca4 βca6*, while *5xβca* represents *βca1 βca2 βca3 βca4 βca6*. (B) Combinations of T-DNA insertions reduce SA and BTH perception. 14-day-old plants were treated with 500 μM SA, 350 μM BTH, or mock solution. One day later, the plants were inoculated with *Pseudomonas syringae* pv. *tomato* isolate DC3000 (*Pto*) at an OD_600_ of 0.1. Three days after inoculation, *Pto* growth was evaluated as the logarithm of colony forming units (cfu) per plant. The remaining *βca* genotypes are showed in S12 Fig. (C) Decrease in effector-triggered immunity. The indicated genotypes were inoculated as in “B” with different *Pto* strains containing the indicated effectors. The numbers after the letters indicate that these are independent experiments and only data with the same number can be compared. The complete set of experiments is showed in S12 Fig. (D) eds-like phenotype. Seven-week-old plants were hand infiltrated with *Pto* at an OD_600_ of 10^−4^. Three days after inoculation, *Pto* growth was evaluated as the logarithm of cfus per g of fresh weight. (E) Decrease in the toxic effect of SA. The *βca* mutants and the controls were grown on MS plates supplied with 0, 200, and 300 μM SA (photographs in S12 Fig), and the chlorophyll contents of the plants were measured as an indication of the response to SA (30 plants in three groups of 10). (F) The *βca* mutants accumulate more SA than wild type. The SA levels (both free and total) were measured three days after mock or *Pto* inoculation as in “B”, with samples of 15 plants in three groups of five.

Since overexpressing some of these βCAs did not produce a measurable phenotype (S10 Fig), we focused on mutants with T-DNA insertions in the *βCA* genes. First, we evaluated the total CA activity in plants lacking a single *βCA*. *βCA1* was the main contributor to CA activity, since its knockout had a strong effect on total activity, whereas knockout of the other *βCAs* had no effect (Fig 6A). In the case of *βCA5*, we examined the offspring of a heterozygous plant for the insertion, since the presence of three independent insertions produced sterile plants in the homozygotes (S11 Fig). When we combined T-DNA insertions in several genes, the total activity decreased considerably, especially in *βca1 βca2 βca4 βca6* and *βca1 βca2 βca3 βca4 βca6* (Fig 6A). We then investigated the SA responses in the *βCA* mutants. We found no difference (in terms of plant growth) in their response to BTH (S12A Fig), but there were differences in the other phenotypes tested.

The accumulation of null alleles led to a decrease in the responses to SA and BTH, as indicated by the difference in bacterial growth in mock- vs. SA (or BTH)-treated plants (Fig 6B and S12 Fig). Different effector triggered immunity (ETI) responses [60] require different components of the SA pathway. Thus, the recognition of avrRpm1, avrRpt2, and avrPphB mainly require NDR1 and RAR1 [61], while avrRps4 mainly requires EDS1, and PAD4 [62], and hopZ1a require none of these [63]. The resultant is that all the ETI tested in this study were compromised (Fig 6C and S12 Fig), therefore the SA pathway is affected in general, not in a subset of the ETI response. Since the quintuple *βCA* mutant phenocopies intermediate *npr1* or *nrb4* alleles, we tested the enhanced disease symptoms (eds) phenotype [9] in this mutant. In two-week-old plants, no difference in bacterial growth was detected between Col-0, *npr1-1*, and the quintuple *βCA* mutant (Fig 6B). However, we also inoculated older (seven-week-old) plants with low levels of inoculum. Under these conditions (eds phenotype, Fig 6D), there was a notable difference in bacterial growth between Col-0 and *npr1-1*, and the phenotype of *βca1 βca2 βca3 βca4 βca6* was intermediate between that of Col-0 and *npr1-1*.

We tested other phenotypes related to SA perception. Thus, the expression of the pathogenesis related protein PR1 is not altered in the quintuple *βca* mutant (S13 Fig). Another phenotype related to SA is plant growth on MS medium containing SA. On this medium, the growth of wild-type plants is reduced compared to control conditions, but the plants remain green. By contrast, *npr1-1* plants are severely affected when grown on MS medium containing SA, exhibiting bleached cotyledons [29]. The *βca1 βca2 βca3 βca4 βca6* plants showed a new phenotype, i.e., this line grew better on MS+SA than did Col-0 (S14 Fig). To quantify this difference, we measured chlorophyll levels in plants grown on 0, 200, and 300 μM SA (Fig 6E), finding marked differences among genotypes. The quintuple *βca* accumulated more SA than wild type in both mock- and *Pto*-infected tissues and when grown on medium containing both free and conjugated SA (Fig 6F), consistent with previous reports that genotypes unable to perceive SA accumulate more endogenous SA than wild type [8][20]. Besides the indicated phenotypes, we saw no obvious phenotypes in these plants and in our conditions (with the exception of *βca5*, S11 Fig)

### Two activities for one protein: CA activity and SA binding

**Fig 7:**
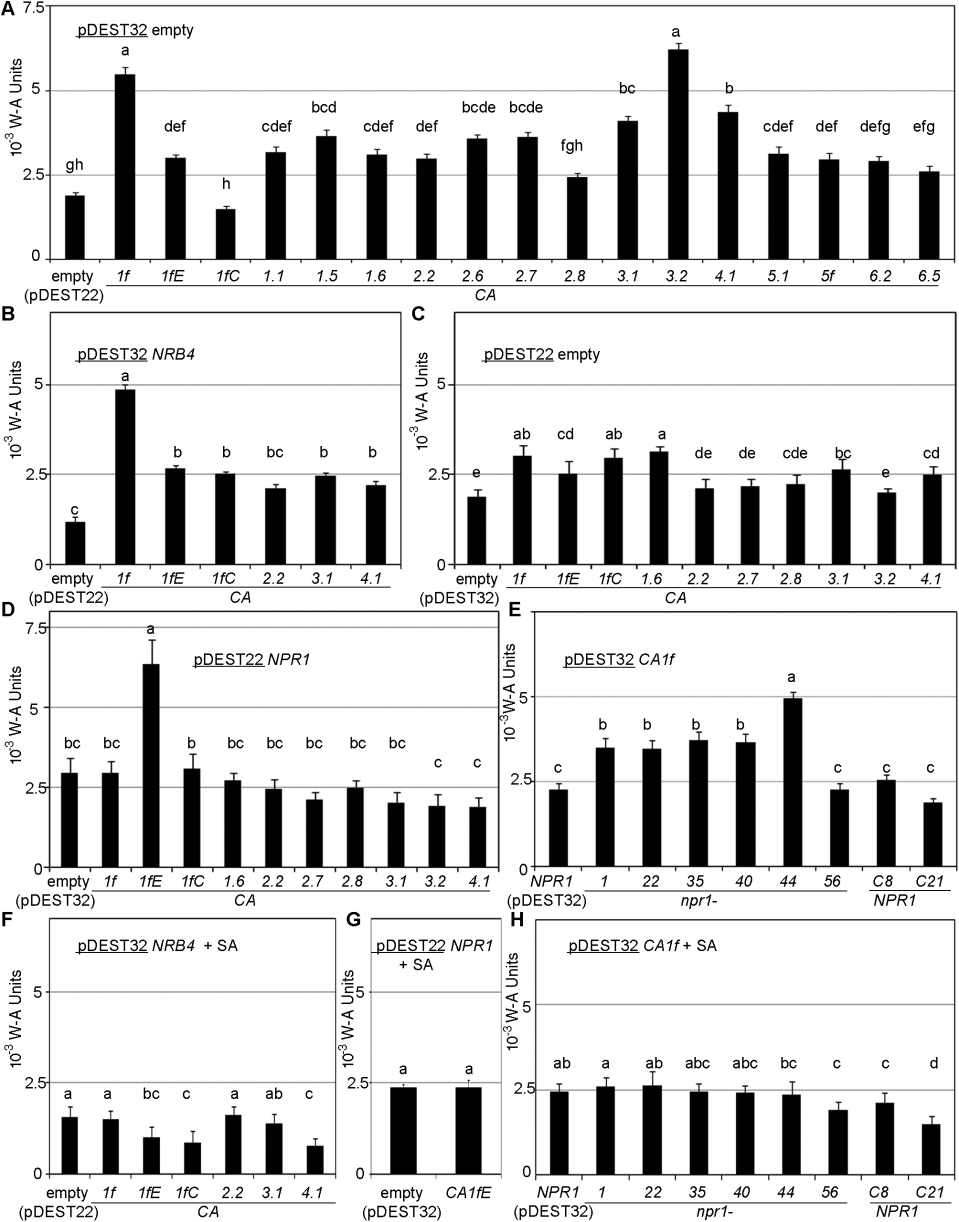
CA activity. CA activity in yeast was measured as in Fig 1F, but with 1 mL of culture (see Methods). (A) Activity of the cloned βCAs in pDEST22 when pDEST32 is empty. *βCA1f*E represents *βCA1f*E110A, and *βCA1f*C represents *βCA1f*C129S. (B) Activity of βCAs in pDEST22 that interact with NRB4 when *NRB4* is present in pDEST32. (C) Activity of βCAs in pDEST32 that interact with NPR1 when pDEST22 is empty. (D) Activity of βCAs in pDEST32 that interact with NPR1 when *NPR1* is present in pDEST22. (E) Activity of βCA1f in pDEST32 when some NPR1 variations are cloned in pDEST22. *NPR1*C8 represents *NPR1*C82A, and *NPR1*C21 represents *NPR1*C216A. (F) Activity of βCAs in pDEST22 that interact with NRB4 when *NRB4* is present in pDEST32, and in the presence of 100 μM SA, compared to “B”. (G) Activity of *βCA1f*E110A cloned in pDEST32, when NPR1 is cloned in pDEST22, and in the presence of SA 100 μM, compared to “D”. (H) Activity of βCA1f in pDEST32 when some NPR1 variations are cloned in pDEST22, and in the presence of SA, compared to “E”.

In addition to functioning in SA perception, βCAs have the enzymatic activity that gives them their name. We therefore measured this activity for each cDNA of the βCAs in yeast. When the βCAs were transformed into yeast in the absence of NRB4 and NPR1, we detected strong variation in CA activity (Fig 7A), with the βCAs that interact with NRB4 or NPR1 showing moderate amounts of CA activity. However, when the assay was performed with βCAs that interact with NRB4 in the presence of this protein, CA activity was reduced for three βCAs, whereas the activity of βCA1f remained unchanged (Fig 7B). Similar results were obtained for NPR1; the βCAs (this time cloned in pDEST32) showed some activity (Fig 7C). However, when NPR1 was present, the activity fell below control levels, except for the mutant βCA1fE, which surprisingly had more activity in the presence of NPR1 than alone (Fig 7D). The interference with CA activity depended on the full functioning of NPR1. Thus, mutations that impair NPR1 functioning *in planta* had no effect on CA activity in βCA1f for five out of six mutants. The point mutations C82A and C216A, which retain NPR1 function, reduced CA activity (Fig 7E). The presence of SA in the medium further reduced CA activity in all cases (Fig 7F, 7G and 7H). Although some CAs also have esterase activity [64], we did not detect such activity using cloned cDNAs (S15 Fig).

**Fig 8:**
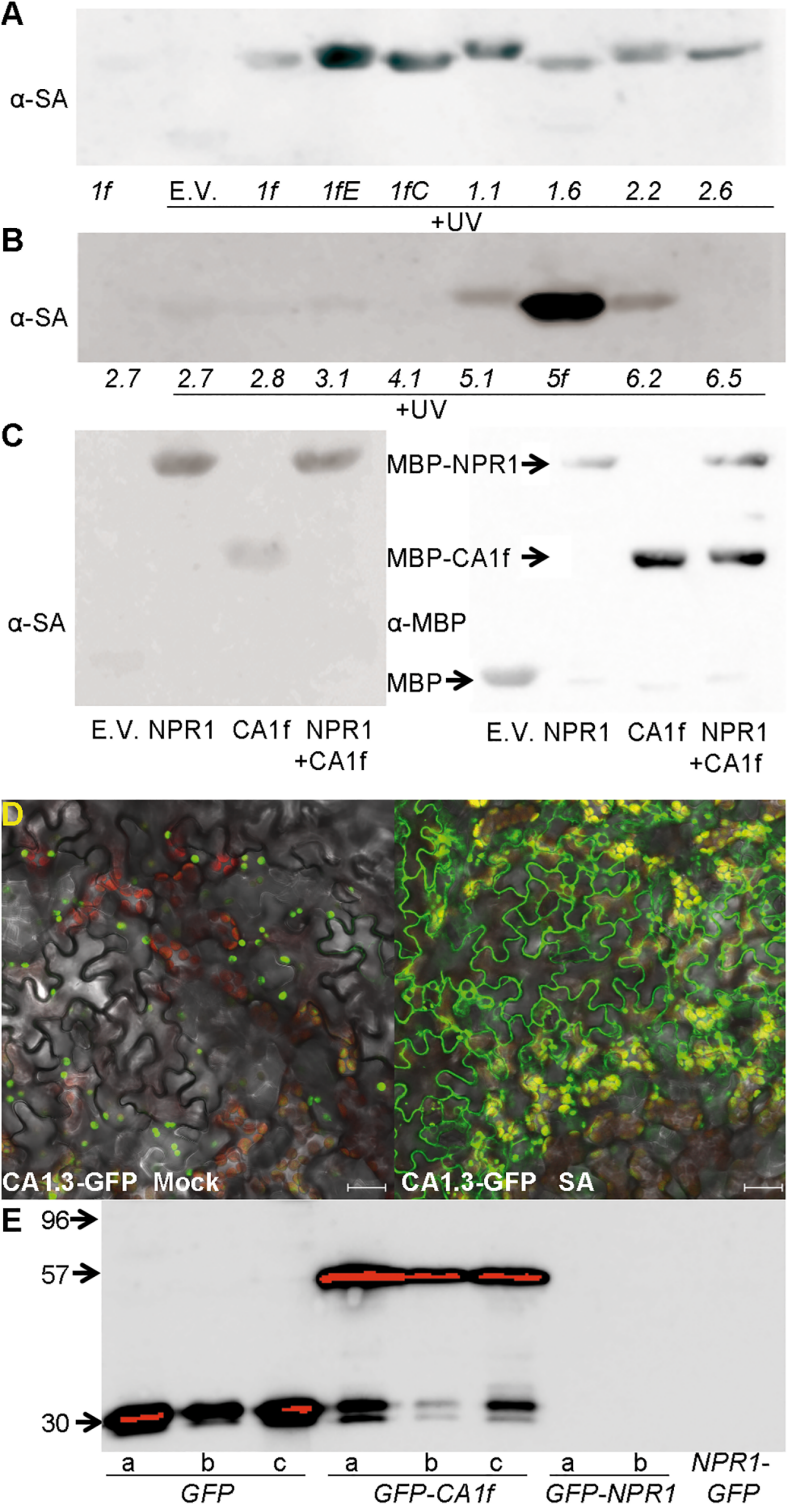
Role of βCAs in SA perception. (A) SA binding of half of the cloned βCAs. Purified recombinant proteins were incubated with 4-AzSA, followed by UV light treatment. 4-AzSA-cross-linked proteins were detected by immunoblot analysis with antibody against SA. The first line corresponds to the negative control, where UV light was omitted. (B) SA binding of the remaining cloned βCAs. (C) Competition for SA binding between βCA1f and NPR1. Similar to “A”, purified recombinant βCA1f and NPR1 proteins were decorated with anti-SA alone or combined. An immunoblot with anti-MBP is shown as a control for protein input. (D) Changes in the localization of βCA1.3-GFP upon SA treatment. Stable transgenic Arabidopsis plants were observed under a confocal microscope one day after treatment. (E) Relative abundance of βCA1f and NPR1 when expressed from the same promoter. Stable transgenic Arabidopsis plants harboring GFP, GFP-βCA1f, and GFP-NPR1 were subject to immunoblot analysis using an anti-GFP antibody. The letters indicate independent lines. In the case of βCA1f, the progeny of a heterozygous plant were analyzed, since no homozygous plant was identified. In the case of NPR1, line a is in the *npr1*-70 background [56], and line b is in the *npr1*-1 background (this work). A *NPR1-GFP* line in the wild-type background [57] was also tested. Except for the *NPR1-GFP* line, the remaining constructs are in the same plasmid backbone, pMDC43. Additional controls for this figure are shown in S15 Fig.

The different βCAs had different affinities for SA (Fig 8A and 8B). Surprisingly, the point mutations constructed in βCA1f increased its affinity for SA. We found a large amount of variation in SA binding among the βCAs examined (βCA1.5 and βCA3.1 could not be expressed in *E*. *coli*), but no clear relationship between SA affinity and protein-protein interactions. βCA5f, which was constructed to remove a putative chloroplastic signal peptide, showed the strongest binding to SA (Fig 8B, additional controls in S15 Fig).

Our results showed that NPR1 and some βCAs interact in yeast and *in planta*. Both proteins bind SA, but NPR1 has an affinity of 140–191 nM [6][12], while that of *Nt*CA1 is 3700 nM [5]. This difference in affinity implies that, in the presence of equal amounts of protein (and if Arabidopsis *βCAs* have a similar affinity than *Nt*CA1), SA would bind to NPR1. We tested this idea (Fig 8C) by repeating the SA binding assay with NPR1 and βCA1f alone and together. When the proteins competed for SA, NPR1 clearly bound to SA. Regarding NRB4, we could not check the interactions with other proteins or SA, since there is no detectable protein in *E*. *coli* or *N*. *benthamiana* [20].

In living plants, proteins are not present in equal amounts; *βCA* mRNAs are expressed at higher levels than *NPR1* mRNA (approximately 30-times higher, S2B Fig). However, some *βCA* mRNA is translated into chloroplastic protein, which would have no opportunity to interact with NPR1. We therefore used confocal microscopy to investigate the behaviors of some of the βCA isoforms fused with GFP. Under mock conditions, βCA1.3-GFP was localized to the chloroplast (Fig 8D, additional controls in S16 Fig), whereas upon SA treatment, it was expressed in the cytosol and chloroplast. Additionally, even when *NPR1* and *βCAs* were expressed from the same promoter, βCA proteins accumulated to higher levels than NPR1. Fig 8E shows the expression patterns of three lines overexpressing *GFP* alone, three lines harboring *GFP-βCA1f*, two lines harboring *GFP-NPR1*, and one line harboring *NPR1-GFP*. GFP and GFP-βCA1f accumulated to high levels, whereas both versions of NPR1 were undetectable (NPR1 was detected using the same construct after transient expression in *N*. *benthamiana*, Fig 5E). Hence, plants contain high levels of βCAs (with low affinity for SA) and very low levels of NPR1 (with high affinity for SA).

## Discussion

### Functions of carbonic anhydrases in plants

The functions of CAs have been highly conserved throughout evolution [23], since all living organisms contain active CAs. Plants have three different families of CAs [27]; we focused on the β family in the current study. Strikingly, the total CA activity in the plant was quite dependent on the β family, since line *βca1 βca2 βca3 βca4 βca6* retained only approximately 10% of total CA activity (Fig 6A).

Several functions have been proposed for CAs. CAs were initially thought to participate in photosynthesis by increasing the amount of CO_2_, which is required by RuBisCo [65]. This hypothesis made sense due to its parallels with the role of CAs in animals, i.e., enhancing the solubility of CO_2_ by converting it to HCO_3_^-^ [23]. However, in C3 plants like Arabidopsis, there is no evidence that CAs play a major role in photosynthesis [66], and the quintuple mutant did not show any visible phenotype under our conditions. Homozygous knockout lines of *βCA5* exhibit smaller, sterile plants, but these plants can perform photosynthesis (S11 Fig). The most obvious potential role for CAs is in the regulation of pH in the cell (mentioned in [66]), but CA mutants also have phenotypes related to lipogenesis [67] and CO_2_ perception. *βCA1* and *βCA4* are required for the response to high CO_2_ involving the stomata [24][68][69].

The interaction between SA and NtCA1 was initially investigated using a biochemical approach, in which chloroplasts were isolated from tobacco and various protein fractions were tested for their ability to bind to SA [5]. Arabidopsis βCA1 can bind to SA [6][26] and is S-nitrosylated upon pathogen infection, which reduces its SA binding and CA activity [26]. Here, we found that SA, its analog BTH, and pathogen infection reduced the CA activity of these proteins, and that plants with elevated levels of SA under mock conditions, such as *npr1-1* and *nrb4-2*, had decreased CA activity (Fig 1F). It is worth to mention than in plants with low levels of SA, as *NahG eds5 sid2*, there is also a decrease in the total CA activity, so the effect of the pathogen is in part independent of SA. This effect is also seen in *npr1-1* and *nrb4-2* (which do not perceive SA) infected with *Pto*, although with these two mutants, the pathogen produce a further increase in SA concentration [20]. Note that the effect of SA in CA activity in Fig 1 is measured globally and they could be explained by gene induction, protein abundance, alteration of catalytic efficiency through complex formation, or presence of an inhibitor. But in yeast, SA treatment also reduced the CA activity conferred by the introduction of individual Arabidopsis *βCAs* (Fig 7F, 7G and 7H). Such an effect was not observed by [5], perhaps because they investigated tobacco rather than Arabidopsis. SA inhibits the CA activity of αCA family members, at least in mammals; perhaps this is a common effect of CAs [70]. When CA activity *in planta* was inhibited by treatment with ethoxyzolamide, the levels of conjugated SA dropped significantly (S1 Fig), revealing a connection between these two activities. When we added the same inhibitors to yeast containing *βCA1f* and *NPR1*, the interaction was not affected (Fig 4D), which argues for the separation of activities for βCA1f (CA activity and SA binding). However, this result appears to contradict the results *in planta*, or perhaps the effect of the inhibitors on all CAs is different from their effect on βCA1f.

### βCAs interact with proteins of the SA pathway

The interaction between NRB4 and βCAs is centered in the KIX domain located at the N-terminal region of NRB4 (Figs 1C, 1D and 4D), where the mutations *nrb4-1*, *nrb4-2*, and *nrb4-3* were found *in planta*, although mutated versions of the protein interacted with the βCAs (Figs 1B and 4D). NRB4 interacted with at least four proteins of the same family, each expressed from a different gene and all independent of the presence of SA in the medium (Figs 2C, 2D and 5B and S7 and S8 Figs). In yeast, there was an additional result: the interactions with NRB4 reduced the CA activity of the βCAs examined (Fig 7A, 7B and 7F). Thus, it appears that by interacting with several βCAs, NRB4 targets these CAs, and the SA bound to them, to the nucleus.

Since four βCAs interacted with NRB4, we reasoned that they might also interact with other proteins of the SA perception pathway. Interestingly, βCA1f did interact with NPR1, in a SA-dependent manner (Fig 3A). This interaction depends on a functional NPR1, since six mutations in NPR1 that rendered the protein inactive *in planta* disrupted the interaction (Fig 3B, [56]), while two mutations that maintained wild-type activity *in planta* did not affect the interaction (Fig 3C, [57]). On the other hand, two mutations in βCA1f that disrupted its CA activity (Fig 7A, [58]) also disrupted the interaction (Fig 3D and S4 Fig), although these mutated proteins bound more SA than wild type (Fig 8A). It is worth mention that some of the negative results in the interactions tested could be due to an unstable fragment of the protein. However, only βCA6.2 and βCA6.5 failed to produce a phenotype in yeast either by interaction or by CA activity (Figs 2, 4 and 7).

Regarding the SA in our experiments, we generally used 100 μM SA (and analogs), which is the same order of magnitude as the concentrations used in other studies [19][71]. Nevertheless, when we tested different SA concentrations, the interaction between and βCA1 and NPR1 increased from 10 μM to 10 mM SA (Fig 4A), providing additional proof of the specificity of the interaction. Clearly, 10 mM is not the physiological SA concentration *in planta*, since a typical SA concentration in a wild-type plant inoculated with *Pto* (when all tissues are pooled) is 0.6 μM, and the level in some mutants can reach 16 μM [20]. However, these values are the average values for all tissues, and it is difficult to determine the true range of SA concentrations inside a plant cell. In any case, the values of SA that allow the interaction between βCA1 and NPR1 are on the order of magnitude of the SA that the proteins likely experience in their cellular location (see below).

The SA analog BTH did not produce the same effect on the βCA1f-NPR1 interaction as SA did (Fig 4B); it is possible that BTH does not enter yeast cells or that BTH is not recognized by the interacting partners. The latter possibility would imply that βCA1f is capable of discriminating between SA and BTH, as NPR1 is known not to discriminate between them [11]. However, the partial loss of SA perception in the quintuple mutant did not lead to discrimination between SA and BTH in terms of pathogen growth (Fig 6B), and thus, the more plausible explanation is that BTH does not enter yeast cells efficiently.

Like NRB4, NPR1 interacted with eight βCAs in yeast (Fig 4E and 4F). We confirmed the interaction with six of these proteins using BiFC (Fig 5D and S7 and S8 Figs) and co-sedimentation assays (Fig 5E and 5F). In all, at least four βCA proteins (βCA1f, βCA2.2, βCA3.1, and βCA4.1) interacted with both NRB4 and NPR1 under all conditions tested (Figs 2, 4 and 5 and S7 Fig). The interactions of NPR1 with the βCAs in yeast also reduced their CA activity considerably (Fig 7C, 7D and 7E). In the absence of an interaction, as with the *npr1* alleles, CA activity is not repressed, with the exception of *npr1*-44. Therefore, in general the interaction between βCA1f and NPR1 or NRB4 inhibits CA activity, and mutations or versions of the proteins that alter the interaction do not alter CA activity. βCAs have been described as having two independent activities, the CA activity and the SA binding [26]. Our results show that the interaction with NPR1 or NRB4 affects the CA activity, which indicates that the active locus is affected in the interaction, either participating in the interaction, or being blocked by it.

### Localizations of the NRB4-βCA and NPR1-βCA interactions

NPR1 is localized to the nucleus and cytosol [72], while NRB4 is localized to the nucleus [20]. Early studies assumed that CAs would localize to the chloroplast [73][74], which would preclude their interaction with cytosolic or nuclear proteins. Recent studies (e.g. [24]), however, were more precise and pointed to a cytosolic localization for the βCAs. At least in the case of *βCA1* and *βCA2*, the genes produce several versions of the proteins, including both cytosolic and chloroplastic forms, some of which interact with NRB4 and NPR1, while others do not (Fig 2B, 2C and 2D and Fig 4E and 4F). In fact, at least in the case of βCA1.3-GFP, treatment with SA altered the localization of the protein, producing a more cytosolic localization (Fig 8D). The signals observed in BiFC for the NRB4-βCA interactions were near, but outside of, the nucleus (Fig 5B and S7A, S8A, S8B and S8C Figs). The interaction of NPR1 with the βCAs also produced some signals localized near and outside the nucleus, but in most cases, the signals were localized inside the nucleus (Fig 5D and S7B and S8D Figs). It is possible that the localization outside the nucleus is an artifact, as the βCAs described here can form octamers *in planta* [23]. If each monomer carries a fragment of the GFP, and the functional reconstitution of GFP stabilizes the interaction (since GFP is very stable, Kerppola, 2009), perhaps the resulting complex (eight βCAs, eight GFPs, eight NPR1 or NRB4) is too large or too stable to completely localize to the nucleus. Notably, while *βCA1* and *βCA2* produce bona fide cytosolic versions, this is not the case with all of the genes. βCA3 has been found in the cytosol [75], but βCA4 is associated with the plasma membrane [69]. βCA5 has been found in the chloroplast [75] although it is also expressed in roots [26], and βCA6 localizes to the mitochondria [75]. Our data do not speak to whether these genes also produce cytosolic versions, since some of our evidence came from transgenic plants expressing only one splicing form. Alternatively, posttranscriptional modifications may occur that would facilitate βCA interactions with NPR1 and/or NRB4. For example, we detected a strong change in the expression and localization of βCA1.3-GFP when SA was applied (Fig 8D). In the case of *βCA1* and *βCA2*, the cytosolic versions (Fig 2B) are produced when an internal ATG is used by the ribosomes, since the mRNA that produces the cytosolic version contains the first ATG, although it is out of frame with the rest of the sequence (TAIR10, www.arabidopsis.org). Therefore, and at least for βCA1, SA appears to target more protein to the cytosol, allowing more interaction with NPR1 and NRB4.

### Knockout lines in the βCAs have various SA perception phenotypes

The βCAs play important roles in SA perception. We observed various phenotypes in several combinations of T-DNA insertion lines, starting from the double *βca1 βca2* mutant up to the quintuple mutant; for simplicity, we will focus on the quintuple mutant. The quintuple mutant is less sensitive to SA and BTH than the wild type, as determined by *Pto* growth (Fig 6B), even though it accumulated more SA in mock and *Pto*-infected tissue (Fig 6F). These and other phenotypes are common in other mutants in SA perception, such as *npr1* [8], *nrb4* [20], and *tga2 tga5 tga6* [17]. However, the βCA mutants have several distinctive phenotypes regarding SA. They are wild type in terms of the weight loss produced by BTH (S10 Fig), and they are wild type in terms of PR1 induction (S13 Fig). The second explanation involves the behavior of the quintuple mutant on MS plates containing SA. While mutants in SA perception were less green than the wild type on this medium, the quintuple mutant was greener than wild type (Fig 6E and S14 Fig). These phenotypes might indicate that βCAs play only a partial role in SA perception, with these phenotypes dependent on *NPR1*, *NRB4*, and *TGAs*, but not on *βCA*s. Alternatively, perhaps no effect of SA is detectable in *βCA5* knockouts (Fig 6A), but the sextuple mutant would be more dramatically affected by SA. Note that a short version of βCA5 had stronger affinity to SA in our assay (Fig 8). A third explanation is that the quantitative effect of mutations in *βCAs* could be strong enough to be detected for some phenotypes, but not others. Weak alleles of *npr1* and *nrb4* (in terms of fresh weight) exhibit almost complete loss of function in terms of pathogen growth [20][28]. Moreover, the loss of SA perception in the quintuple mutant could occur just at the right level to make MS-SA plates less toxic.

A role for *βCAs* in defense has been reported previously, since silencing of a *CA* gene in potato increased the rate of infection by *Phytophthora infestans* [76], while silencing this gene in tobacco reduced the effect of the *Pto-avrPto* interaction [5]. In Arabidopsis, βCA1 is S-nitrosylated in response to pathogen attack, which decreases its binding to SA; in addition, a T-DNA insertion in *βCA1* increases the susceptibility of plants to *Pto* (*avrB*) [26]. βCA1 was also found to interact with an effector from *Hyaloperonospora arabidopsidis* (HaRxLL470_WACO9, [77]). While these studies were performed using a single gene or cDNA, the current results were obtained using different combinations of β-family members. Since there is such redundancy in the family of βCAs, only when two or more genes are knocked out the total CA activity is seriously compromised (Fig 6). Our approach using combinations of knockouts in the βCA family allowed us to explore the role in of this family in SA perception and defense.

### The role of βCAs in SA perception

How do βCAs take part in the perception of SA? The βCAs bind to SA, resulting in the downregulation of CA activity (Figs 1F, 7F, 7G and 7H). The CAs were proposed to function as transponders or senzymes [78], with important roles not only in CO_2_ fixation, but also in CO_2_ signaling *in planta* ([69] and references therein). Thus, the reduction in CA activity could affect multiple targets, such as lipid production [67] and cellular pH [66], without affecting the transcription of defense genes. Indeed, there are examples of other signals that are perceived and act in the cytosol, without nuclear intervention. In fact, some proteins that bind SA are thought to function in this fashion [6]. A non-exclusive alternative is that the βCAs could help bring SA to NPR1. The expression levels of *βCAs* were approximately 30-times that of *NPR1* (S2 Fig), and βCAs accumulated too much higher levels than NPR1 when expressed from the same promoter (Fig 8E. We include an additional NPR1-GFP [57] as a control. Note that these three NPR1 lines produce enough protein to complement a mutant). Therefore, it is likely that SA interacts with βCAs before interacting with NPR1 in the cytosol. As βCAs themselves interact with NPR1, SA would likely then bind NPR1, since the affinity of NPR1 for SA is approximately 20 times stronger, and the βCAs would be taken to the nucleus by NPR1. In the absence of βCAs (e.g., in the quintuple mutant), SA is still perceived by NPR1, but less efficiently (Fig 6). NPR1 binds to transcription factors in the nucleus to fulfill their functions [16], and NRB4, a likely paralog of MED15 localized in the nucleus, could connect NPR1 (bound to transcription factors and βCAs) with RNA Pol II [21]. Overall, our results suggest that βCAs are quite important, although not strictly required, for SA perception.

## Supporting information

S1 Fig Relationship between SA and CA activity. (A) Arabidopsis plants, ecotype Col-0 were treated with 100 μM of the CA inhibitors acetazolamide (AA), ethoxyzolamide (EZ), sulfanilamide (SU), or mock solution. One day later, the amounts of free and total SA (free plus glucoside conjugated) were measured as described in the Methods. (B) In the same experiment, samples were taken to measure CA activity as described in the Methods. Note that acetazolamide and sulfanilamide do not cross membranes, while ethoxyzolamide does, thereby inhibiting CAs *in vivo*.
(PDF)

S2 Fig Microarray data from public repositories. (A) Data for the six *βCAs*, *NPR1*, and *NRB4* were downloaded from BAR (Version 14–05; http://bar.utoronto.ca). *βCA1*, *βCA2*, *βCA4*, and *βCA5* are repressed by pathogen infection, while *βCA3* and *βCA6* are induced. Note that the scale is different in different graphs. (B) Relative expression of the six *βCAs*, *NPR1*, and *NRB4*. The data were downloaded from TAIR (www.arabidopsis.org). All the data available was used, regardless of the age or tissue. The graph on the left shows the expression of *βCA1*, *βCA2*, and *βCA4*, since their expression levels were higher, and the graph on the right shows the expression of the remaining *βCAs*, along with *NPR1* and *NRB4*. (C) Expression of the *βCAs* 24 hours after BTH application. The data (E-GEOD-10646) were downloaded from ArrayExpress (www.ebi.ac.uk/arrayexpress/). (D) Expression of the *βCAs* in an *npr1*-1 background (E-GEOD-5745). (E) Expression of the *βCAs* in an *nrb4*-2 and *nrb4*-4 background (E-MEXP-3602).
(PDF)

S3 Fig Alignment of βCA protein sequences. The amino acid sequences of the proteins described in Fig 2B were aligned with Lasergene MegAlign Pro software from DNASTAR, Inc. (Madison, WI, USA). All the CAs are from the β family.
(PDF)

S4 Fig Mutations of βCA1f. (A) The βCA1f sequence is shown with mutations E110A and C129S. (B) Controls for βCA1fC129S in yeast. βCA1fC129S showed some autoactivation in the yeast two-hybrid system (Fig 3). In this photograph, the same yeast strains were grown in medium containing 5 mM 3AT, showing that there is no interaction between NPR1 and βCA1fC129S that could be concealed by the autoactivation of βCA1fC129S.
(PDF)

S5 Fig Additional controls for Fig 4. SA and the analogs used in Fig 4 do not affect the growth or basal activity of yeast. (A) Effects of SA and analogs on a strain of yeast containing empty pDONR22 and pDONR32. (B) Effects of SA and BTH on a strain of yeast containing NPR1 in pDONR22, showing that there is no measureable increase in activity. (C) The CA inhibitors only minimally affect the interaction. The CA inhibitors acetazolamide (AA), ethoxyzolamide (EZ), and sulfanilamide (SU) were added to the liquid culture, alone or with SA, and the interaction was quantified as described in Fig 4. (D) Yeast three hybrid. No interaction was detected when βCA1f was cloned in pARC352 and introduced into yeast harboring NRB4 and NPR1 cloned in the indicated plasmids.
(PDF)

S6 Fig SA measurements in *N*. *benthamiana* after transient expression. *N*. *benthamiana* samples were taken four days after infiltration with *Agrobacterium tumefaciens* with no plasmids (Empty Agro.), *Agrobacterium* with plasmids (*35S*:*NPR1*, *35S*:*NRB4*, and *35S*:*βCA1f*, Agro. with Plasmids), or no treatment (Mock). (A) In two out of four experiments, an increase in SA content was not detected. (B) In the two other experiments, the concentrations of free and total SA increased.
(PDF)

S7 Fig All βCAs that interacted *in planta* with NRB4 and NPR1. (A) Interaction of NRB4 with βCAs. The photograph at the left shows a negative interaction of NRB4 with βCA2.8 (as a control) by bimolecular fluorescence complementation (BiFC), while the remaining photographs show positive interactions (detectable GFP) with βCA1f, βCA2.2, βCA3.1 and βCA4.1. (B) Interaction of NPR1 with βCAs. Similarly, the photograph on the left shows a negative BiFC interaction of NPR1 with βCA3.2, and the remaining photographs show a positive interaction with βCA1f, βCA2.2, βCA3.1, βCA4.1, βCA5.1, and βCA6.2. The white bars represent 20 μm.
(PDF)

S8 Fig Details and magnified views of BiFC. (A) DAPI staining of the interaction between NRB4-βCA2.2. (B) Detailed view of the NRB4-βCA1f interaction. (C) Detailed view of the NRB4-βCA3.1 interaction. (D) Detailed view of the NPR1-βCA1f interaction. (E) Triple interaction NPR1-βCA1f-NRB4. The photograph on the left shows a negative interaction of NRB4 with NPR1 when a third empty vector is added. (F) Positive interaction of NRB4-NPR1 in the presence of βCA1f. The signal is weak; yellow arrows point to nuclei where GFP is visible. (G) Magnified view of the nucleus indicated by the top yellow arrow. (H) Magnified view of the nucleus indicated by the middle yellow arrow. (I) Magnified view of the nucleus indicated by the yellow arrow at the bottom.
(PDF)

S9 Fig Controls for co-sedimentations. (A) *GFP*-*NPR1* and various *MBP*-*βCAs* were transiently expressed in *N*. *benthamiana* by agroinfiltration and pulled-down with amylose resin. The panel shows *GFP*-NPR1 detected by immunoblot before treatment with resin. In the case of the empty vector, the image was cut to show that the lane developed over a shorter period of time. Ponceau-S staining of the nitrocellulose membrane is shown as a loading control. (B) The equivalent experiment, one day after treatment with 1 mM SA. (C) Expression of βCAs-MBP detected by immunoblot analysis before treatment with resin. (D) The equivalent experiment, one day after treatment with 1 mM SA. (E) Co-sedimentation of NPR1 with βCAs after purification with amylose resin; the sedimented fractions were decorated with the indicated antibodies. (F) The equivalent experiment, one day after treatment with 1 mM SA.
(PDF)

S10 Fig Phenotypes of the transgenic βCA lines. Selected cDNAs from βCAs were transformed into Arabidopsis under the control of the 35S promoter and fused to GFP. (A) N-terminal fusions; the cDNAs of proteins that interact with both NPR1 and NRB4 were selected, and the response of the transgenic lines to BTH was measured in terms of weight, as in Fig 2A. (B) Response of the N-terminal fusions to SA and BTH in terms of *Pto* growth. Asterisks indicate statistically significant differences from the mock treatment (P < 0.05 one asterisk, P < 0.01 two) using the Student’s t test (one tail). (C) C-terminal fusions. Representative cDNAs of each gene were selected due to their chloroplastic or unknown localizations, and the response of the transgenic lines to BTH was measured. (D) Response of the C-terminal fusions to SA and BTH in terms of *Pto* growth. Three independent, homozygous lines were selected for each cDNA. In the case of βCA1f, no homozygous line could be recovered, and the progeny of one transgenic plant per line were used.
(PDF)

S11 Fig The *βca5* mutant is sterile. (A) Phenotypes of *βca5* plants. The progeny of a heterozygous *βca5-3* plant were sown individually, and the photograph was taken four weeks later. Of the 30 plants in the photograph, seven are homozygous for the T-DNA insertion in βCA5, corresponding to the smaller plants. These smaller plants were later checked using PCR markers and found to be homozygous. (B) Close-up view of a homozygous *βca5-3* plant, corresponding to the plant in the bottom left corner of “A”. (C) The homozygous *βca5-3* plants bolt, but the flowers do not produce fruits. (D) Close-up view of a fertile, heterozygous *βca5-3* flower. (E) Close-up view of a homozygous *βca5-3* flower.
(PDF)

S12 Fig Additional phenotypes of the T-DNA insertion lines (I). (A) The response of the βCAs null alleles and their combinations to BTH was measured in terms of weight, as in Fig 2A. (B) Response of the null alleles to SA and BTH in terms of *Pto* growth. Fig 6B shows only the most important genotypes of this experiment. (C) Response to *Pto(avrRpm1)*. (D) Response to *Pto(avrRpt2)*. (E) Response to *Pto(avrPphB)*. (F) Response to *Pto(avrRps4)*. (G) Response to *Pto(hopZ1a)*. Fig 6C shows condensed information from panels “C” to “G”.
(PDF)

S13 Fig Induction of PR1 in Col-0 and in the quintuple mutant. Immunoblot analysis with anti-PR1 [28], in Col-0 (A) and the quintuple βca mutant (B) after 1 mM SA treatment. Samples were taken before, and at the indicated days after the spray.
(PDF)

S14 Fig Additional phenotypes of the T-DNA insertion lines (II). (A) The single T-DNA insertion lines were grown on MS plates, and the photograph was taken at day 7 after germination. (B) The same genotypes as in “A”, grown on MS with 300 μM SA. (C) Lines with multiple T-DNA insertions grown on MS plates. (D) The same genotypes as in “C”, grown on MS with 300 μM SA. Quantification of the response of *βca1 βca2 βca3 βca4 βca6* (*5x βca*) and its controls is shown in Fig 6E.
(PDF)

S15 Fig Additional controls for Fig 8. (A) SA binding of half of the cloned βCAs. Purified recombinant proteins were incubated with 4-AzSA, followed by UV light treatment; 4-AzSA-cross-linked proteins were detected by immunoblot analysis with antibody against SA. An immunoblot with anti-MBP is shown as a control for protein input. The first line corresponds to a negative control where UV light was omitted. (B) SA binding of the remaining cloned βCAs. (C) Behavior of GFP alone under mock and SA conditions. Note that GFP is localized to the cytosol and nucleus (not chloroplast), and there is no change upon SA treatment. (D) Changes in the localization of βCA1.3-GFP upon SA treatment. Stable transgenic Arabidopsis plants were observed under a confocal microscope one day after treatment. (E) Relative abundance of βCA1f and NPR1 when expressed from the same promoter. Stable transgenic Arabidopsis plants harboring GFP, GFP-βCA1f, and GFP-NPR1 were subject to immunoblot analysis using an anti-GFP antibody. The letters indicate independent lines. In the case of βCA1f, the progeny of a heterozygous plant were analyzed, since no homozygous plants were identified. In the case of NPR1, line “a” is in the *npr1-70* background [56], and line “b” is in the *npr1-1* background (this work). A *NPR1-GFP* line in the WT background was also tested [57]. Except for the *NPR1-GFP* line, the remaining constructs are in the same plasmid backbone, pMDC43. Ponceau staining is shown below as a loading control.
(PDF)

S16 Fig Esterase activity of the βCAs. Esterase activity of the cloned βCAs. Esterase activity was determined as described [64]. Serial dilutions of commercial esterase (Ref 75742 SIGMA) were included as an internal control.
(PDF)

S1 Table Primers used in this study. (PDF)
