# Supplementary material for: β-carbonic anhydrases play a role in salicylic acid perception in Arabidopsis
Source: PLoS One. 2017 Jul 28;12(7):e0181820. doi: 10.1371/journal.pone.0181820 (PMC5533460; doi:10.1371/journal.pone.0181820)
Supplement: S1 Fig — (A) Arabidopsis plants, ecotype Col-0 were treated with 100 μM of the CA inhibitors acetazolamide (AA), ethoxyzolamide (EZ), sulfanilamide (SU), or mock solution. One day later, the amounts of free and total SA (free plus glucoside conjugated) were measured as described in the Methods. (B) In the same experiment, samples were taken to measure CA activity as described in the Methods. Note that acetazolamide and sulfanilamide do not cross membranes, while ethoxyzolamide does, thereby inhibiting CAs in vivo. (PDF) [file pone.0181820.s001.pdf]

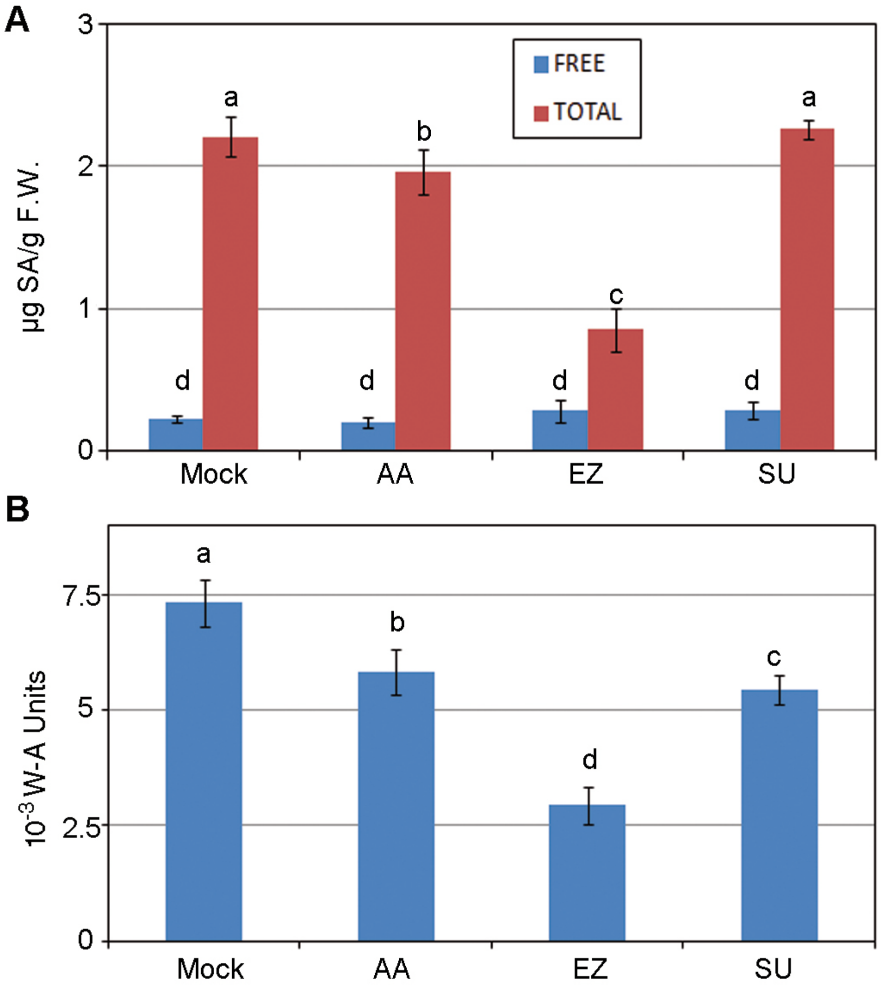

S1 Fig. Relationship between SA and CA activity. (A) Arabidopsis plants, ecotype Col-0 were treated with 100 µM of the CA inhibitors acetazolamide (AA), ethoxyzolamide (EZ), sulfanilamide (SU), or mock solution. One day later, the amounts of free and total SA (free plus glucoside conjugated) were measured as described in the Methods. (B) In the same experiment, samples were taken to measure CA activity as described in the Methods. Note that acetazolamide and sulfanilamide do not cross membranes, while ethoxyzolamide does, thereby inhibiting CAs *in vivo*.
