# Supplementary material for: β-carbonic anhydrases play a role in salicylic acid perception in Arabidopsis
Source: PLoS One. 2017 Jul 28;12(7):e0181820. doi: 10.1371/journal.pone.0181820 (PMC5533460; doi:10.1371/journal.pone.0181820)
Supplement: S2 Fig — (A) Data for the six βCAs, NPR1, and NRB4 were downloaded from BAR (Version 14–05; http://bar.utoronto.ca). βCA1, βCA2, βCA4, and βCA5 are repressed by pathogen infection, while βCA3 and βCA6 are induced. Note that the scale is different in different graphs. (B) Relative expression of the six βCAs, NPR1, and NRB4. The data were downloaded from TAIR (www.arabidopsis.org). All the data available was used, regardless of the age or tissue. The graph on the left shows the expression of βCA1, βCA2, and βCA4, since their expression levels were higher, and the graph on the right shows the expression of the remaining βCAs, along with NPR1 and NRB4. (C) Expression of the βCAs 24 hours after BTH application. The data (E-GEOD-10646) were downloaded from ArrayExpress (www.ebi.ac.uk/arrayexpress/). (D) Expression of the βCAs in an npr1-1 background (E-GEOD-5745). (E) Expression of the βCAs in an nrb4-2 and nrb4-4 background (E-MEXP-3602). (PDF) [file pone.0181820.s002.pdf]

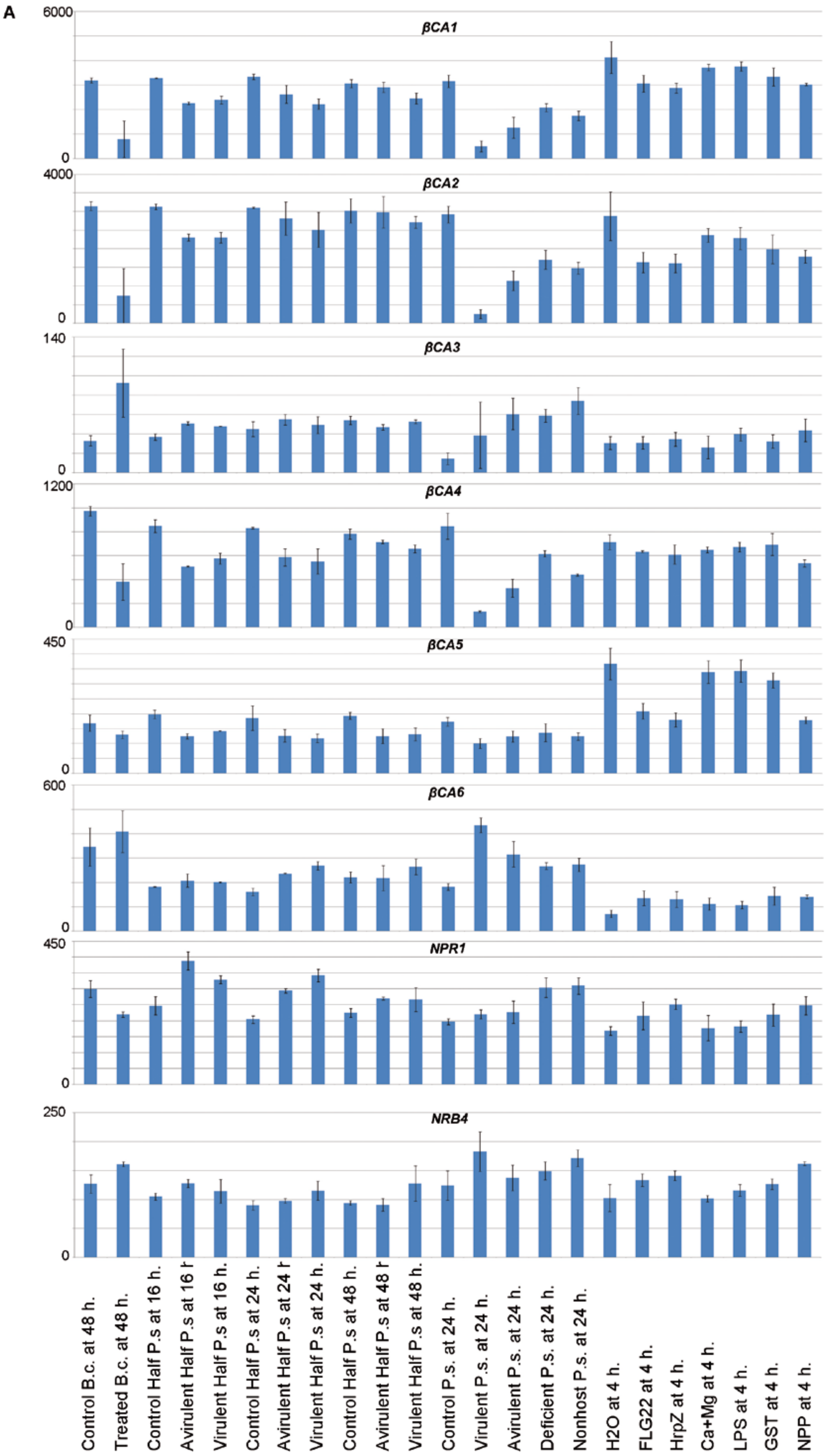
A
βCA1
6000
0
βCA2
4000
0
βCA3
140
0
βCA4
1200
0
βCA5
450
0
βCA6
600
0
NPR1
450
0
NRB4
250
0
Control B.c. at 48 h.
Treated B.c. at 48 h.
Control Half P.s at 16 h.
Avirulent Half P.s at 16 h
Virulent Half P.s at 16 h.
Control Half P.s at 24 h.
Avirulent Half P.s at 24 h
Virulent Half P.s at 24 h.
Control Half P.s at 48 h.
Avirulent Half P.s at 48 h
Virulent Half P.s at 48 h.
Control P.s. at 24 h.
Virulent P.s. at 24 h.
Avirulent P.s. at 24 h.
Deficient P.s. at 24 h.
Nonhost P.s. at 24 h.
H2O at 4 h.
FLG22 at 4 h.
HrpZ at 4 h.
Ca+Mg at 4 h.
LPS at 4 h.
GST at 4 h.
NPP at 4 h.

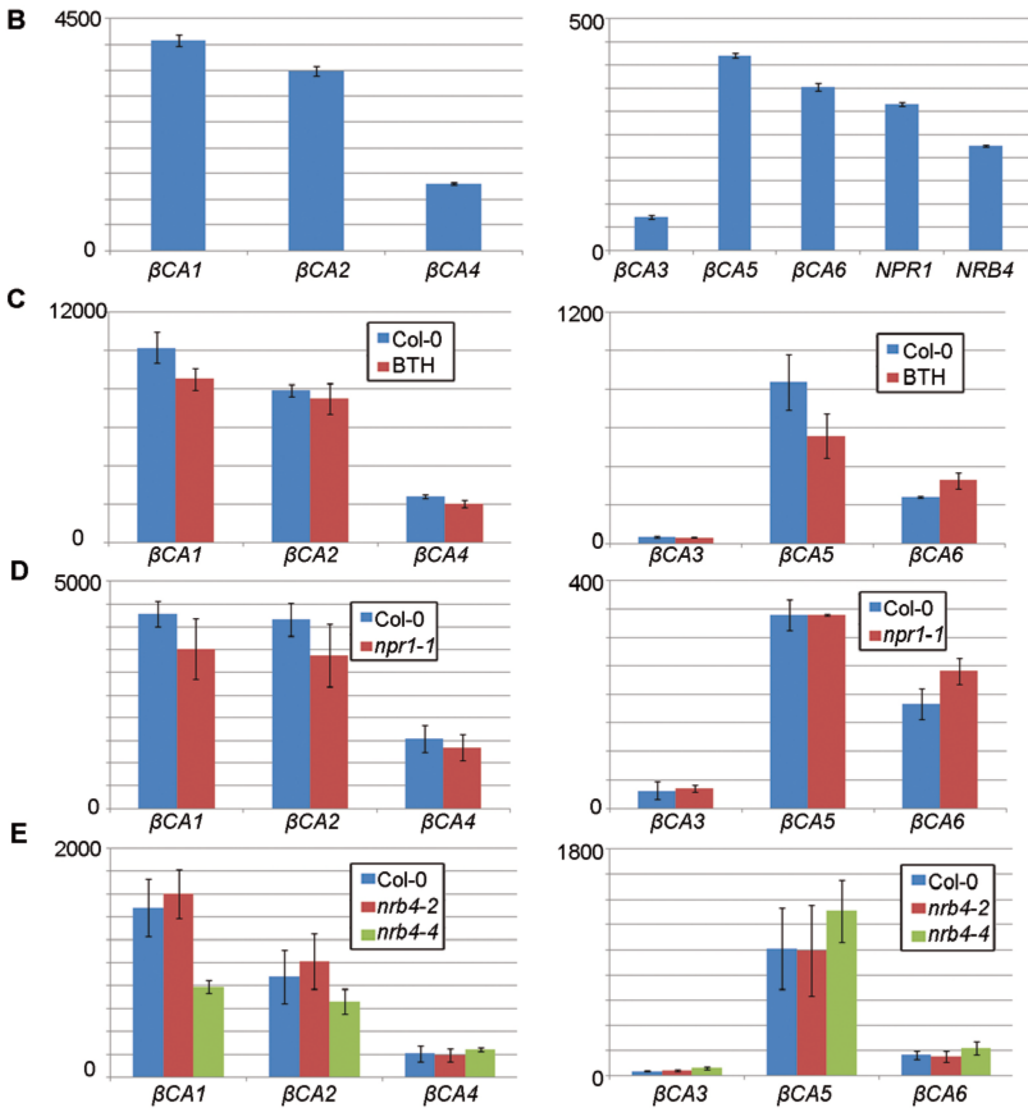

S2 Fig. Microarray data from public repositories. (A) Data for the six *βCAs*, *NPR1*, and *NRB4* were downloaded from BAR (Version 14-05; http://bar.utoronto.ca). *βCA1, βCA2, βCA4*, and *βCA5* are repressed by pathogen infection, while *βCA3* and *βCA6* are induced. Note that the scale is different in different graphs. (B) Relative expression of the six *βCAs*, *NPR1*, and *NRB4*. The data were downloaded from TAIR (www.arabidopsis.org). All the data available was used, regardless of the age or tissue. The graph on the left shows the expression of *βCA1*, *βCA2*, and *βCA4*, since their expression levels were higher, and the graph on the right shows the expression of the remaining *βCAs*, along with *NPR1* and *NRB4*. (C) Expression of the *βCAs* 24 hours after BTH application. The data (E-GEOD-10646) were downloaded from ArrayExpress (www.ebi.ac.uk/arrayexpress/). (D) Expression of the *βCAs* in an *npr1*-1 background (E-GEOD-5745). (E) Expression of the *βCAs* in an *nrb4*-2 and *nrb4*-4 background (E-MEXP-3602).
