# Supplementary material for: β-carbonic anhydrases play a role in salicylic acid perception in Arabidopsis
Source: PLoS One. 2017 Jul 28;12(7):e0181820. doi: 10.1371/journal.pone.0181820 (PMC5533460; doi:10.1371/journal.pone.0181820)
Supplement: S3 Fig — The amino acid sequences of the proteins described in Fig 2B were aligned with Lasergene MegAlign Pro software from DNASTAR, Inc. (Madison, WI, USA). All the CAs are from the β family. (PDF) [file pone.0181820.s003.pdf]

```
          ------------------------------------------------------------
                   10        20        30        40        50        60

CA1f      ------------------------------------------------------------ 1
CA1_1     ------------------------------------------------------------ 1
CA1_3     MSTAPLSGFFLTSLSPSQSSLQKLSLRTSSTVACLPPASSSSSSSSSSSSSRSVP-TLIRN 59
CA1_4     MSTAPLSGFFLTSLSPSQSSLQKLSLRTSSTVACLPPASSSSSSSSSSSSSRSVP-TLIRN 59
CA1_5     ------------------------------------------------------------ 1
CA1_6     ------------------------------------------------------------ 1
CA2f      ------------------------------------------------------------ 1
CA2.1     ------MVPFWTTVSRNGSSDSETTLQSASKATKQYKYPSLRPSHRLSLLFLFPFHLSAN 54
CA2.2     ------------------------------------------------------------ 1
CA2.3     ------MVPFWTTVSRNGSSDSETTLQSASKATKQYKYPSLRPSHRLSLLFLFPFHLSAN 54
CA2.4     ------MVPFWTTVSRNGSSDSETTLQSASKATKQYKYPSLRPSHRLSLLFLFPFHLSAN 54
CA2.6     ------------------------------------------------------------ 1
CA2.7     ------------------------------------------------------------ 1
CA2.8     ------------------------------------------------------------ 1
CA3.1     ------------------------------------------------------------ 1
CA3.2     ------------------------------------------------------------ 1
CA4.1     ------------------------------------------------------------ 1
CA5.1     -------------------------------------------------MAATPTHFSVS 11
CA5f      ------------------------------------------------------------ 1
CA6.2     -------------------------------------------------MAFTLGGRARR 11
CA6.5     ------------------------------------------------------------ 1
```

```
          ------------------MGTESYEDAIEALKKLLIEKD-DLKDVAAAKVKKITAELQAA
                   70        80        90       100       110       120

CA1f      -------------------------------------------TVAAAKVEQITAALQTG 17
CA1_1     ------------------MGTEAYDEAIEALKKLLIEKE-ELKTVAAAKVEQITAALQTG 41
CA1_3     EPVFAAPAPIIAPYWSEEMGTEAYDEAIEALKKLLIEKE-ELKTVAAAKVEQITAALQTG 118
CA1_4     EPVFAAPAPIIAPYWSEEMGTEAYDEAIEALKKLLIEKE-ELKTVAAAKVEQITAALQTG 118
CA1_5     ------------------MGTEAYDEAIEALKKLLIEKE-ELKTVAAAKVEQITAALQTG 41
CA1_6     ------------------MGTEAYDEAIEALKKLLIEKE-ELKTVAAAKVEQITAALQTG 41
CA2f      ---------------------ESYEDAIEALKKLLIEKD-DLKDVAAAKVKKITAELQAA 38
CA2.1     GACFRCTCFSHFKLELRRMGNESYEDAIEALKKLLIEKD-DLKDVAAAKVKKITAELQAA 113
CA2.2     ------------------MGNESYEDAIEALKKLLIEKD-DLKDVAAAKVKKITAELQAA 41
CA2.3     GACFRCTCFSHFKLELRRMGNESYEDAIEALKKLLIEKD-DLKDVAAAKVKKITAELQAA 113
CA2.4     GACFRCTCFSHFKLELRRMGNESYEDAIEALKKLLIEKD-DLKDVAAAKVKKITAELQAA 113
CA2.6     ------------------MGNESYEDAIEALKKLLIEKD-DLKDVAAAKVKKITAELQAA 41
CA2.7     ------------------MGNESYEDAIEALKKLLIEKD-DLKDVAAAKVKKITAELQAA 41
CA2.8     ------------------MGNESYEDAIEALKKLLIEKD-DLKDVAAAKVKKITAELQAA 41
CA3.1     ------------------MSTESYEDAIKRLGELLSKKS-DLGNVAAAKIKKLTDELEEL 41
CA3.2     ------------------MSTESYEAAIKRLGELLSKKS-DLGNVAAAKIKKLTDELEEL 41
CA4.1     ------------------MATESYEAAIKGLNDLLSTKA-DLGNVAAAKIKALTAELKEL 41
CA5.1     HDPFSSTSLLNLQTQAIFGPNHSLKTTQLRIPASFRRKATNLQVMASGKTPGLTQEANGV 71
CA5f      --------------------------------------------MASGKTPGLTQEANGV 16
CA6.2     LVSATSVHQNGCLHKLQQIGSDRFQLGEAKAIRLLPRRT------NMVQELGIREEFMDL 65
CA6.5     ------------------------------MPTLYKKAG------FMVQELGIREEFMDL 24
```

```
                 S S S D S K S F D P V E R I K E G F V T F K K E K Y E T N P A L Y G E L A K G Q S P K Y M V F A C S D S R V C P S H V L
                          130       140       150       160       170       180

CA1f     T S S D K K A F D P V E T I K Q G F I K F K K E K Y E T N P A L Y G E L A K G Q S P K Y M V F A C S D S R V C P S H V L  77
CA1_1    T S S D K K A F D P V E T I K Q G F I K F K K E K Y E T N P A L Y G E L A K G Q S P K Y M V F A C S D S R V C P S H V L  101
CA1_3    T S S D K K A F D P V E T I K Q G F I K F K K E K Y E T N P A L Y G E L A K G Q S P K Y M V F A C S D S R V C P S H V L  178
CA1_4    T S S D K K A F D P V E T I K Q G F I K F K K E K Y E T N P A L Y G E L A K G Q S P K Y M V F A C S D S R V C P S H V L  178
CA1_5    T S S D K K A F D P V E T I K Q G F I K F K K E K Y E T N P A L Y G E L A K G Q S P K Y M V F A C S D S R V C P S H V L  101
CA1_6    T S S D K K A F D P V E T I K Q G F I K F K K E K Y E T N P A L Y G E L A K G Q S P K Y M V F A C S D S R V C P S H V L  101
CA2f     S S S D S K S F D P V E R I K E G F V T F K K E K Y E T N P A L Y G E L A K G Q S P K Y M V F A C S D S R V C P S H V L  98
CA2.1    S S S D S K S F D P V E R I K E G F V T F K K E K Y E T N P A L Y G E L A K G Q S P K Y M V F A C S D S R V C P S H V L  173
CA2.2    S S S D S K S F D P V E R I K E G F V T F K K E K Y E T N P A L Y G E L A K G Q S P K Y M V F A C S D S R V C P S H V L  101
CA2.3    S S S D S K S F D P V E R I K E G F V T F K K E K Y E T N P A L Y G E L A K G Q S P K Y M V F A C S D S R V C P S H V L  173
CA2.4    S S S D S K S F D P V E R I K E G F V T F K K E K Y E T N P A L Y G E L A K G Q S P K Y M V F A C S D S R V C P S H V L  173
CA2.6    S S S D S K S F D P V E R I K E G F V T F K K E K Y E T N P A L Y G E L A K G Q S P K Y M V F A C S D S R V C P S H V L  101
CA2.7    S S S D S K S F D P V E R I K E G F V T F K K E K Y E T N P A L Y G E L A K G Q S P K Y M V F A C S D S R V C P S H V L  101
CA2.8    S S S D S K S F D P V E R I K E G F V T F K K E K Y E T N P A L Y G E L A K G Q S P K Y M V F A C S D S R V C P S H V L  101
CA3.1    D S N K L - - - D A V E R I K S G F L H F K T N N Y E K N P T L Y N S L A K S Q T P K F L V F A C A D S R V S P S H I L  98
CA3.2    D S N K L - - - D A V E R I K S G F L H F K T N N Y E K N P T L Y N S L A K S Q T P K F L V F A C A D S R V S P S H I L  98
CA4.1    D S S N S - - - D A I E R I K T G F T Q F K T E K Y L K N S T L F N H L A K T Q T P K F L V F A C S D S R V C P S H I L  98
CA5.1    A I D R Q N N T D V F D D M K Q R F L A F K K L K Y M D D F E H Y K N L A D A Q A P K F L V I A C A D S R V C P S A V L  131
CA5f     A I D R Q N N T D V F D D M K Q R F L A F K K L K Y M D D F E H Y K N L A D A Q A P K F L V I A C A D S R V C P S A V L  76
CA6.2    N R E T E T S Y D F L D E M R H R F L K F K R Q K Y L P E I E K F K A L A I A Q S P K V M V I G C A D S R V C P S Y V L  125
CA6.5    N R E T E T S Y D F L D E M R H R F L K F K R Q K Y L P E I E K F K A L A I A Q S P K V M V I G C A D S R V C P S Y V L  84

                 D F Q P G D A F V V R N I A N M V P P F D K V K Y A G V G A A I E Y A V L H L K V E N I V V I G H S A C G G I K G L M S
                          190       200       210       220       230       240

CA1f     D F Q P G D A F V V R N I A N M V P P F D K V K Y G G V G A A I E Y A V L H L K V E N I V V I G H S A C G G I K G L M S  137
CA1_1    D F Q P G D A F V V R N I A N M V P P F D K V K Y G G V G A A I E Y A V L H L K V E N I V V I G H S A C G G I K G L M S  161
CA1_3    D F Q P G D A F V V R N I A N M V P P F D K V K Y G G V G A A I E Y A V L H L K V E N I V V I G H S A C G G I K G L M S  238
CA1_4    D F Q P G D A F V V R N I A N M V P P F D K V K Y G G V G A A I E Y A V L H L K V E N I V V I G H S A C G G I K G L M S  238
CA1_5    D F Q P G D A F V V R N I A N M V P P F D K V K Y G G V G A A I E Y A V L H L K V E N I V V I G H S A C G G I K G L M S  161
CA1_6    D F Q P G D A F V V R N I A N M V P P F D K V K Y G G V G A A I E Y A V L H L K V E N I V V I G H S A C G G I K G L M S  161
CA2f     D F H P G D A F V V R N I A N M V P P F D K V K Y A G V G A A I E Y A V L H L K V E N I V V I G H S A C G G I K G L M S  158
CA2.1    D F H P G D A F V V R N I A N M V P P F D K V K Y A G V G A A I E Y A V L H L K V E N I V V I G H S A C G G I K G L M S  233
CA2.2    D F H P G D A F V V R N I A N M V P P F D K V K Y A G V G A A I E Y A V L H L K V E N I V V I G H S A C G G I K G L M S  161
CA2.3    D F H P G D A F V V R N I A N M V P P F D K V K Y A G V G A A I E Y A V L H L K V E N I V V I G H S A C G G I K G L M S  233
CA2.4    D F H P G D A F V V R N I A N M V P P F D K V K Y A G V G A A I E Y A V L H L K V E N I V V I G H S A C G G I K G L M S  233
CA2.6    D F H P G D A F V V R N I A N M V P P F D K V K Y A G V G A A I E Y A V L H L K V E N I V V I G H S A C G G I K G L M S  161
CA2.7    D F H P G D A F V V R N I A N M V P P F D K V K Y A G V G A A I E Y A V L H L K V E N I V V I G H S A C G G I K G L M S  161
CA2.8    D F H P G D A F V V R N I A N M V P P F D K V K Y A G V G A A I E Y A V L H L K V E N I V V I G H S A C G G I K G L M S  161
CA3.1    N F Q L G E A F I V R N I A N M V P P Y D K T K H S N V G A A L E Y P I T V L N V E N I L V I G H S C C G G I K G L M A  158
CA3.2    N F Q L G E A F I V R N I A N M V P P Y D K T K H S N V G A A L E Y P I T V L N V E N I L V I G H S C C G G I K G L M A  158
CA4.1    N F Q P G E A F V V R N I A N M V P P F D Q K R H S G V G A A V E Y A V V H L K V E N I L V I G H S C C G G I K G L M S  158
CA5.1    G F Q P G D A F T V R N I A N L V P P Y E S - G P T E T K A A L E F S V N T L N V E N I L V I G H S R C G G I Q A L M K  190
CA5f     G F Q P G D A F T V R N I A N L V P P Y E S - G P T E T K A A L E F S V N T L N V E N I L V I G H S R C G G I Q A L M K  135
CA6.2    G F Q P G E A F T I R N V A N L V T P V Q N - G P T E T N S A L E F A V T T L Q V E N I I V M G H S N C G G I A A L M S  184
CA6.5    G F Q P G E A F T I R N V A N L V T P V Q N - G P T E T N S A L E L R S P L F R L R T L                                  127
```

```
             FPLDGN-NSTDFIEDWVKICLPAKSKVLAESEDSAFEDQCGRCEREAVNVSLANLLTYPF
                       250       260       270       280       290       300

CA1f         FPLDGN-NSTDFIEDWVKICLPAKSKVISELGDSAFEDQCGRCEREAVNVSLANLLTYPF 196
CA1_1        FPLDGN-NSTDFIEDWVKICLPAKSKVISELGDSAFEDQCGRCEREAVNVSLANLLTYPF 220
CA1_3        FPLDGN-NSTDFIEDWVKICLPAKSKVISELGDSAFEDQCGRCEREAVNVSLANLLTYPF 297
CA1_4        FPLDGN-NSTDFIEDWVKICLPAKSKVISELGDSAFEDQCGRCER-----------VYKH 286
CA1_5        FPLDGN-NSTDFIEDWVKICLPAKSKVISELGDSAFEDQCGRCEREAVNVSLANLLTYPF 220
CA1_6        FPLDGN-NSTDFIEDWVKICLPAKSKVISELGDSAFEDQCGRCER-----------VYKH 209
CA2f         FPLDGN-NSTDFIEDWVKICLPAKSKVLAESESSAFEDQCGRCEREAVNVSLANLLTYPF 217
CA2.1        FPLDGN-NSTDFIEDWVKICLPAKSKVLAESESSAFEDQCGRCEREAVNVSLANLLTYPF 292
CA2.2        FPLDGN-NSTDFIEDWVKICLPAKSKVLAESESSAFEDQCGRCEREAVNVSLANLLTYPF 220
CA2.3        FPLDGN-NSTDFIEDWVKICLPAKSKVLAESESSAFEDQCGRCER-VLNVSLANLLTYPF 291
CA2.4        FPLDGN-NSTDFIEDWVKICLPAKSKVLAESESSAFEDQCGRCER-AVNVSLANLLTYPF 291
CA2.6        FPLDGN-NSTDFIEDWVKICLPAKSKVLAESESSAFEDQCGRCEREAVNVSLANLLTYPF 220
CA2.7        FPLDGN-NSTDFIEDWVKICLPAKSKVLAESESSAFEDQCGRCER-VLNVSLANLLTYPF 219
CA2.8        FPLDGN-NSTDFIEDWVKICLPAKSKVLAESESSAFEDQCGRCER-AVNVSLANLLTYPF 219
CA3.1        IEDNTAPTKTEFIENWIQICAPAKNRIKQDCKDLSFEDQCTNCEKEAVNVSLGNLLSYPF 218
CA3.2        IEDNTAPTKTEFIENWIQICAPAKNRIKQDCKDLSFEDQCTNCEKEAVNVSLGNLLSYPF 218
CA4.1        IEDDAAPTQSDFIENWVKIGASARNKIKEEHKDLSYDDQCNKCEKEAVNVSLGNLLSYPF 218
CA5.1        ME-DEG-DSRSFIHNWVVVGKKAKESTKAVASNLHFDHQCQHCEKASINHSLERLLGYPW 248
CA5f         ME-DEG-DSRSFIHNWVVVGKKAKESTKAVASNLHFDHQCQHCEKASINHSLERLLGYPW 193
CA6.2        HQNHQG-QHSSLVERWVMNGKAAKLRTQLASSHLSFDEQCRNCEKESIKDSVMNLITYSW 243
CA6.5                                                                     127

             VREGVVKGTLALKGGYYDFVNGSFELWELQFGISP-------VHSI-----------
                       310       320       330       340       350

CA1f         VREGLVKGTLALKGGYYDFVKGAFELWGLEFGLSE-------TSSV              235
CA1_1        VREGLVKGTLALKGGYYDFVKGAFELWGLEFGLSE-------TSSVKDVATILHWKL   270
CA1_3        VREGLVKGTLALKGGYYDFVKGAFELWGLEFGLSE-------TSSV              336
CA1_4        MLDR                                                         290
CA1_5        VREGLVKGTLALKGGYYDFVKGAFELWGLEFGLSE-------TSSV              259
CA1_6        MLDR                                                         213
CA2f         VREGVVKGTLALKGGYYDFVNGSFELWELQFGISP-------VHSI              256
CA2.1        VREGVVKGTLALKGGYYDFVNGSFELWELQFGISP-------VHSI              331
CA2.2        VREGVVKGTLALKGGYYDFVNGSFELWELQFGISP-------VHSI              259
CA2.3        VREGVVKGTLALKGGYYDFVNGSFELWELQFGISP-------VHSI              330
CA2.4        VREGVVKGTLALKGGYYDFVNGSFELWELQFGISP-------VHSI              330
CA2.6        VREGVVKGTLALKGGYY--ET---------QLSCTK-------LAL              248
CA2.7        VREGVVKGTLALKGGYYDFVNGSFELWELQFGISP-------VHSI              258
CA2.8        VREGVVKGTLALKGGYYDFVNGSFELWELQFGISP-------VHSI              258
CA3.1        VRERVVKNKLAIRGAHYDFVKGTFDLWELDFKTTP-------AFALS             258
CA3.2        VRERVVKNKLAIRGAHYDFVKGTFDLWELDFKTTP-------AFALS             258
CA4.1        VRAEVVKNTLAIRGGHYNFVKGTFDLWELDFKTTP-------AFAFS             258
CA5.1        IEEKVRQGSLSLHGGYYNFVDCTFEKWTVDYAASRGKKKEGSGIAVKDRSVWS       301
CA5f         IEEKVRQGSLSLHGGYYNFVDCTFEKWTVDYAASRGKKKEGSGIAVKDRSVWS       246
CA6.2        IRDRVKRGEVKIHGCYYNLSDCSLEKWRLSSDKTN------YGFYISDREIWS       290
CA6.5                                                                     127
```

S3 Fig. Alignment of βCA protein sequences. The amino acid sequences of the proteins described in Fig 2B were aligned with Lasergene MegAlign Pro software from DNASTAR, Inc. (Madison, WI, USA). All the CAs are from the β family.
