# Supplementary material for: β-carbonic anhydrases play a role in salicylic acid perception in Arabidopsis
Source: PLoS One. 2017 Jul 28;12(7):e0181820. doi: 10.1371/journal.pone.0181820 (PMC5533460; doi:10.1371/journal.pone.0181820)
Supplement: S4 Fig — (A) The βCA1f sequence is shown with mutations E110A and C129S. (B) Controls for βCA1fC129S in yeast. βCA1fC129S showed some autoactivation in the yeast two-hybrid system (Fig 3). In this photograph, the same yeast strains were grown in medium containing 5 mM 3AT, showing that there is no interaction between NPR1 and βCA1fC129S that could be concealed by the autoactivation of βCA1fC129S. (PDF) [file pone.0181820.s004.pdf]

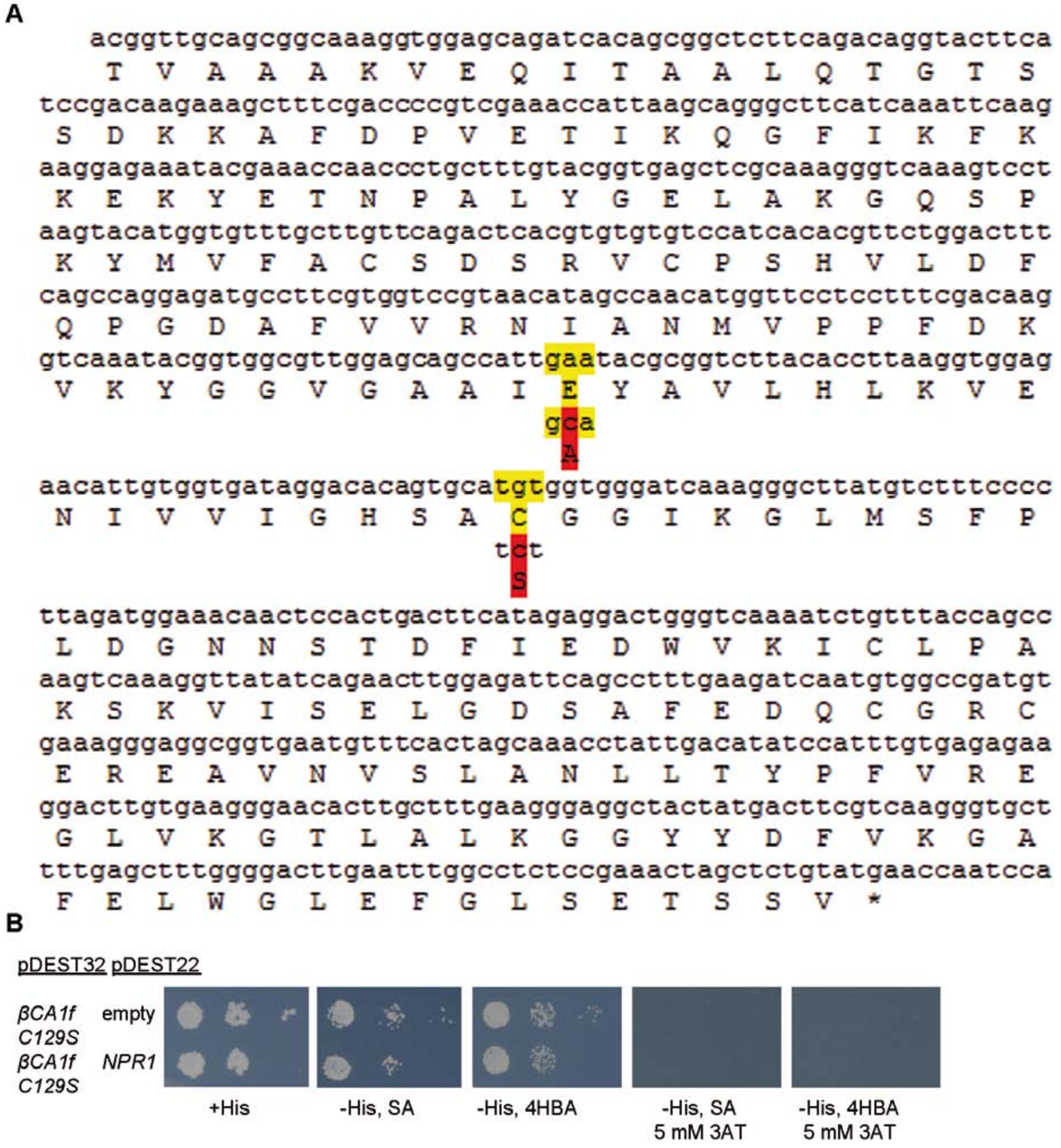

S4 Fig. Mutations of βCA1f. (A) The βCA1f sequence is shown with mutations E110A and C129S. (B) Controls for βCA1fC129S in yeast. βCA1fC129S showed some autoactivation in the yeast two-hybrid system (Fig 3). In this photograph, the same yeast strains were grown in medium containing 5 mM 3AT, showing that there is no interaction between NPR1 and βCA1fC129S that could be concealed by the autoactivation of βCA1fC129S.
