# Supplementary material for: β-carbonic anhydrases play a role in salicylic acid perception in Arabidopsis
Source: PLoS One. 2017 Jul 28;12(7):e0181820. doi: 10.1371/journal.pone.0181820 (PMC5533460; doi:10.1371/journal.pone.0181820)
Supplement: S5 Fig — SA and the analogs used in Fig 4 do not affect the growth or basal activity of yeast. (A) Effects of SA and analogs on a strain of yeast containing empty pDONR22 and pDONR32. (B) Effects of SA and BTH on a strain of yeast containing NPR1 in pDONR22, showing that there is no measureable increase in activity. (C) The CA inhibitors only minimally affect the interaction. The CA inhibitors acetazolamide (AA), ethoxyzolamide (EZ), and sulfanilamide (SU) were added to the liquid culture, alone or with SA, and the interaction was quantified as described in Fig 4. (D) Yeast three hybrid. No interaction was detected when βCA1f was cloned in pARC352 and introduced into yeast harboring NRB4 and NPR1 cloned in the indicated plasmids. (PDF) [file pone.0181820.s005.pdf]

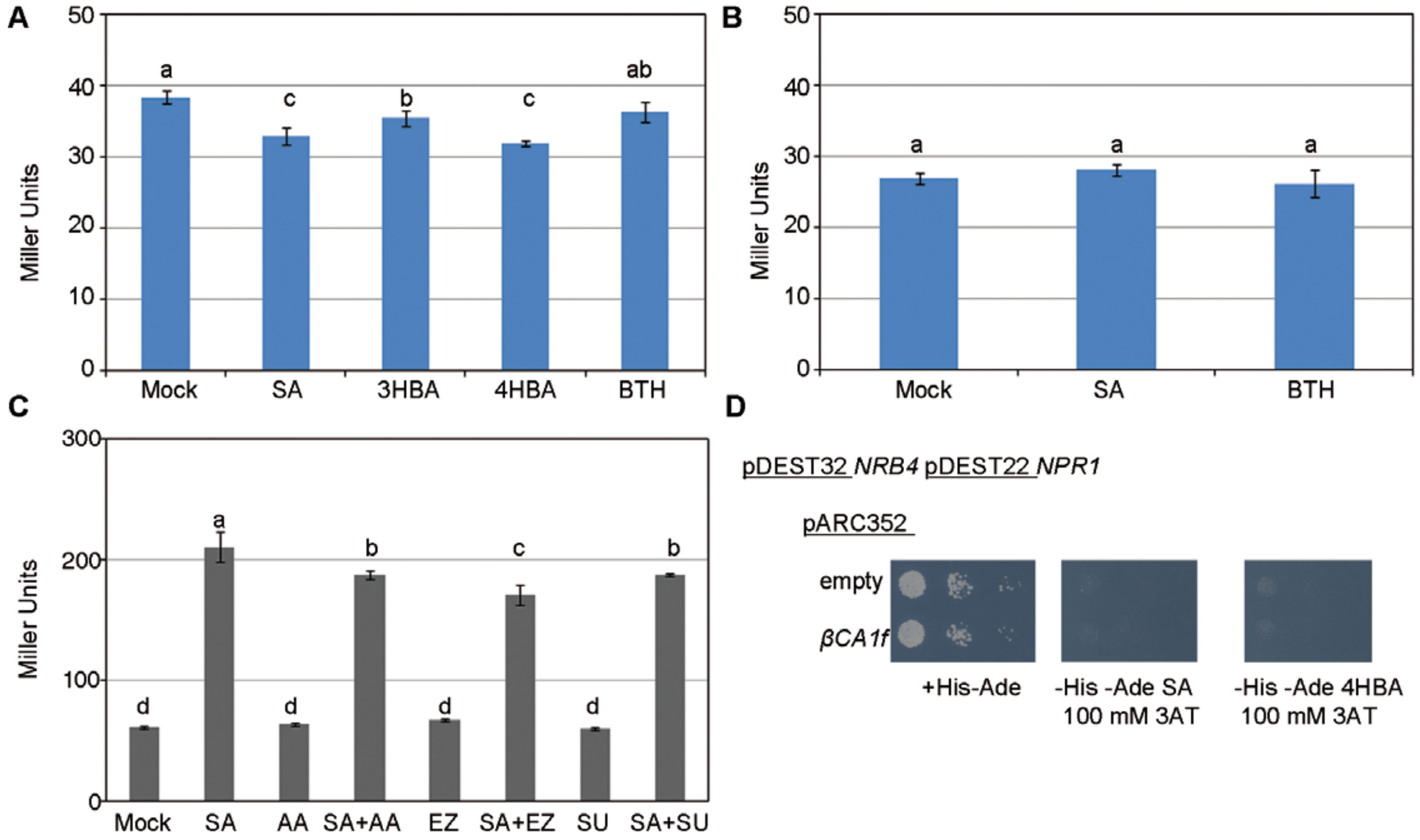

S5 Fig. Additional controls for Fig 4. SA and the analogs used in Fig 4 do not affect the growth or basal activity of yeast. (A) Effects of SA and analogs on a strain of yeast containing empty pDONR22 and pDONR32. (B) Effects of SA and BTH on a strain of yeast containing NPR1 in pDONR22, showing that there is no measureable increase in activity. (C) The CA inhibitors only minimally affect the interaction. The CA inhibitors acetazolamide (AA), ethoxyzolamide (EZ), and sulfanilamide (SU) were added to the liquid culture, alone or with SA, and the interaction was quantified as described in Fig 4. (D) Yeast three hybrid. No interaction was detected when βCA1f was cloned in pARC352 and introduced into yeast harboring NRB4 and NPR1 cloned in the indicated plasmids.
