# Supplementary material for: β-carbonic anhydrases play a role in salicylic acid perception in Arabidopsis
Source: PLoS One. 2017 Jul 28;12(7):e0181820. doi: 10.1371/journal.pone.0181820 (PMC5533460; doi:10.1371/journal.pone.0181820)
Supplement: S6 Fig — N. benthamiana samples were taken four days after infiltration with Agrobacterium tumefaciens with no plasmids (Empty Agro.), Agrobacterium with plasmids (35S:NPR1, 35S:NRB4, and 35S:βCA1f, Agro. with Plasmids), or no treatment (Mock). (A) In two out of four experiments, an increase in SA content was not detected. (B) In the two other experiments, the concentrations of free and total SA increased. (PDF) [file pone.0181820.s006.pdf]

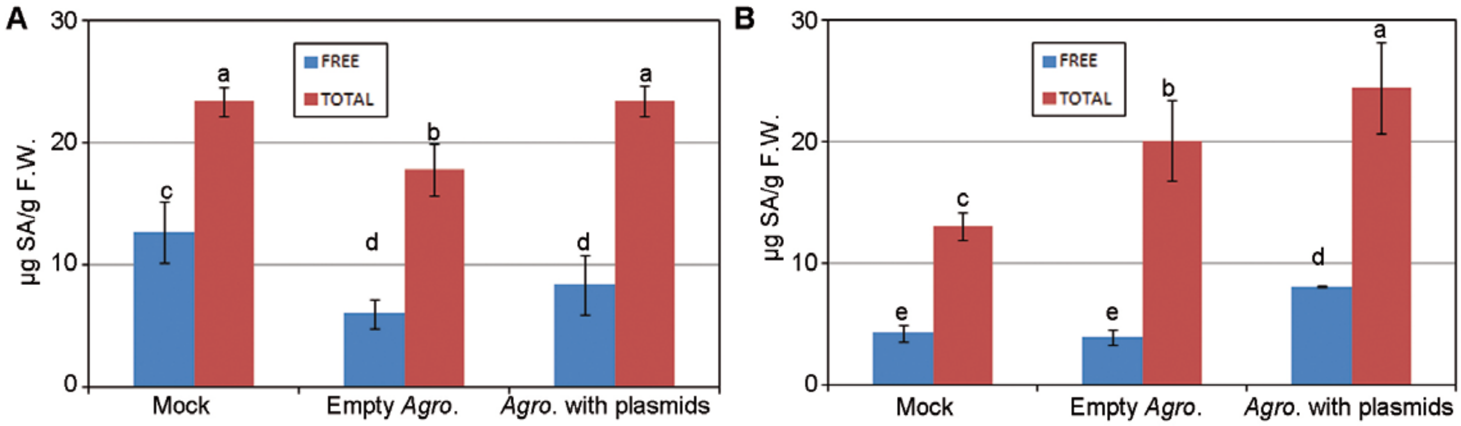


**S6 Fig. SA measurements in *N. benthamiana* after transient expression.** *N. benthamiana* samples were taken four days after infiltration with *Agrobacterium tumefaciens* with no plasmids (Empty Agro.), *Agrobacterium* with plasmids (*35S:NPR1*, *35S:NRB4*, and *35S:βCA1f*, Agro. with Plasmids), or no treatment (Mock). (A) In two out of four experiments, an increase in SA content was not detected. (B) In the two other experiments, the concentrations of free and total SA increased.
