# Supplementary material for: β-carbonic anhydrases play a role in salicylic acid perception in Arabidopsis
Source: PLoS One. 2017 Jul 28;12(7):e0181820. doi: 10.1371/journal.pone.0181820 (PMC5533460; doi:10.1371/journal.pone.0181820)
Supplement: S7 Fig — (A) Interaction of NRB4 with βCAs. The photograph at the left shows a negative interaction of NRB4 with βCA2.8 (as a control) by bimolecular fluorescence complementation (BiFC), while the remaining photographs show positive interactions (detectable GFP) with βCA1f, βCA2.2, βCA3.1 and βCA4.1. (B) Interaction of NPR1 with βCAs. Similarly, the photograph on the left shows a negative BiFC interaction of NPR1 with βCA3.2, and the remaining photographs show a positive interaction with βCA1f, βCA2.2, βCA3.1, βCA4.1, βCA5.1, and βCA6.2. The white bars represent 20 μm. (PDF) [file pone.0181820.s007.pdf]

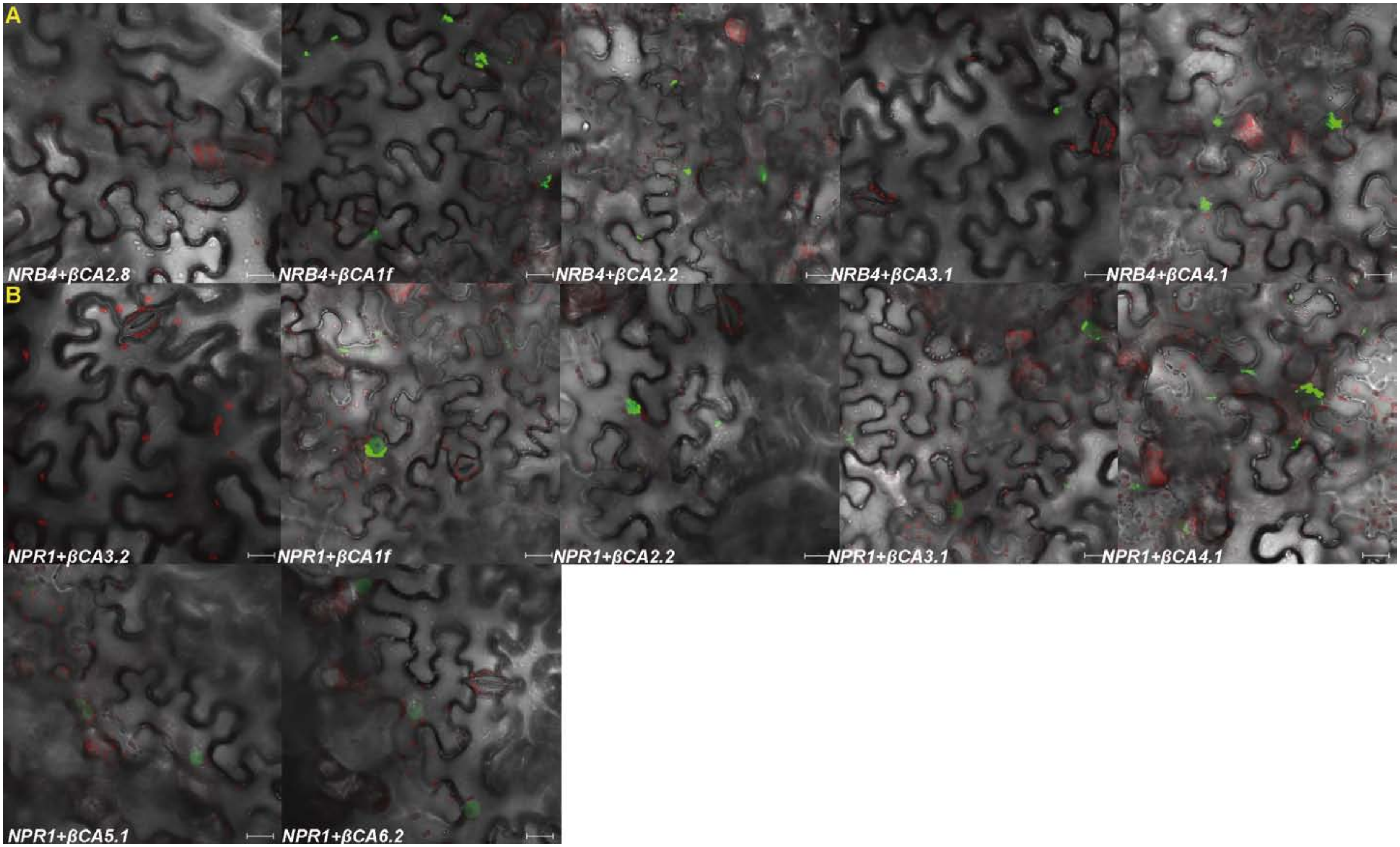
A
NRB4+βCA2.8
NRB4+βCA1f
NRB4+βCA2.2
NRB4+βCA3.1
NRB4+βCA4.1
B
NPR1+βCA3.2
NPR1+βCA1f
NPR1+βCA2.2
NPR1+βCA3.1
NPR1+βCA4.1
NPR1+βCA5.1
NPR1+βCA6.2

S7 Fig. All βCAs that interacted *in planta* with NRB4 and NPR1. (A) Interaction of NRB4 with βCAs. The photograph at the left shows a negative interaction of NRB4 with βCA2.8 (as a control) by bimolecular fluorescence complementation (BiFC), while the remaining photographs show positive interactions (detectable GFP) with βCA1f, βCA2.2, βCA3.1 and βCA4.1. (B) Interaction of NPR1 with βCAs. Similarly, the photograph on the left shows a negative BiFC interaction of NPR1 with βCA3.2, and the remaining photographs show a positive interaction with βCA1f, βCA2.2, βCA3.1, βCA4.1, βCA5.1, and βCA6.2. The white bars represent 20 µm.
