# Supplementary material for: β-carbonic anhydrases play a role in salicylic acid perception in Arabidopsis
Source: PLoS One. 2017 Jul 28;12(7):e0181820. doi: 10.1371/journal.pone.0181820 (PMC5533460; doi:10.1371/journal.pone.0181820)
Supplement: S8 Fig — (A) DAPI staining of the interaction between NRB4-βCA2.2. (B) Detailed view of the NRB4-βCA1f interaction. (C) Detailed view of the NRB4-βCA3.1 interaction. (D) Detailed view of the NPR1-βCA1f interaction. (E) Triple interaction NPR1-βCA1f-NRB4. The photograph on the left shows a negative interaction of NRB4 with NPR1 when a third empty vector is added. (F) Positive interaction of NRB4-NPR1 in the presence of βCA1f. The signal is weak; yellow arrows point to nuclei where GFP is visible. (G) Magnified view of the nucleus indicated by the top yellow arrow. (H) Magnified view of the nucleus indicated by the middle yellow arrow. (I) Magnified view of the nucleus indicated by the yellow arrow at the bottom. (PDF) [file pone.0181820.s008.pdf]

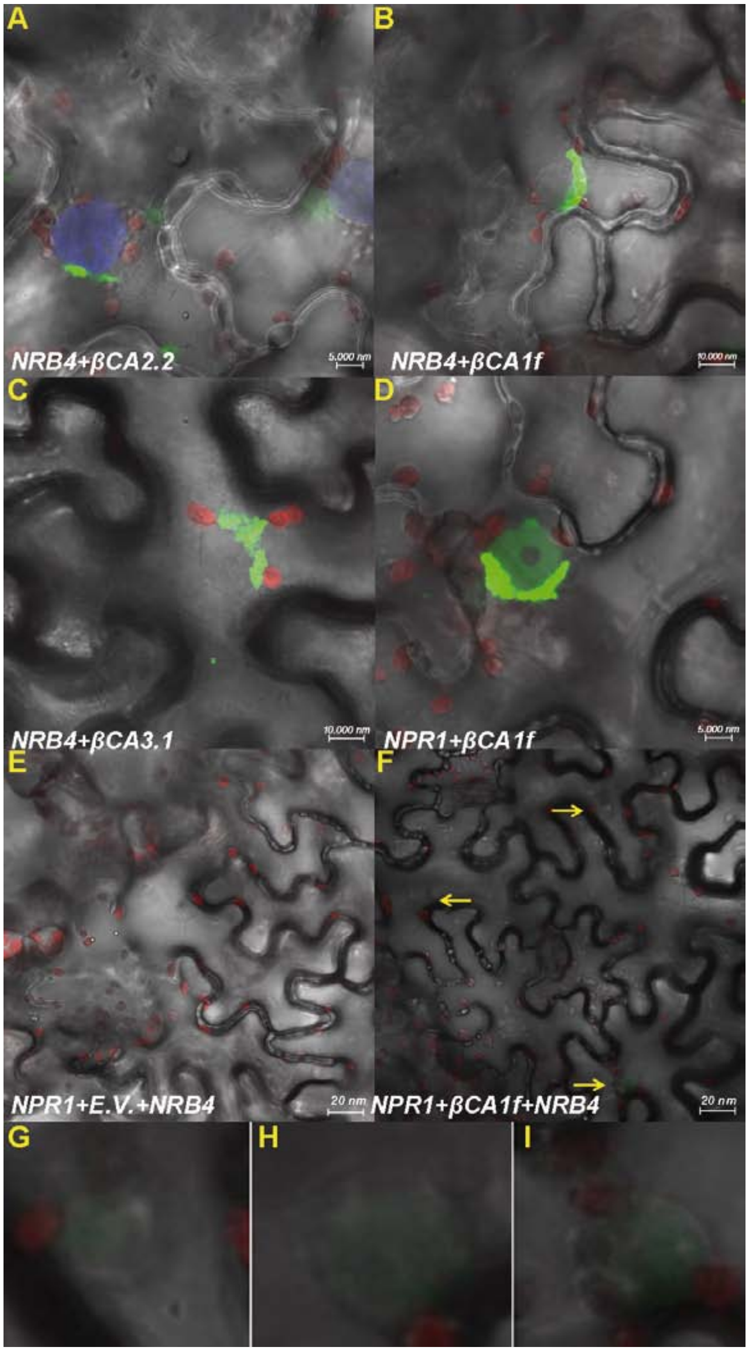

S8 Fig. Details and magnified views of BiFC. (A) DAPI staining of the interaction between NRB4-βCA2.2. (B) Detailed view of the NRB4-βCA1f interaction. (C) Detailed view of the NRB4-βCA3.1 interaction. (D) Detailed view of the NPR1-βCA1f interaction. (E) Triple interaction NPR1-βCA1f-NRB4. The photograph on the left shows a negative interaction of NRB4 with NPR1 when a third empty vector is added. (F) Positive interaction of NRB4-NPR1 in the presence of βCA1f. The signal is weak; yellow arrows point to nuclei where GFP is visible. (G) Magnified view of the nucleus indicated by the top yellow arrow. (H) Magnified view of the nucleus indicated by the middle yellow arrow. (I) Magnified view of the nucleus indicated by the yellow arrow at the bottom.
