# Supplementary material for: β-carbonic anhydrases play a role in salicylic acid perception in Arabidopsis
Source: PLoS One. 2017 Jul 28;12(7):e0181820. doi: 10.1371/journal.pone.0181820 (PMC5533460; doi:10.1371/journal.pone.0181820)
Supplement: S9 Fig — (A) GFP-NPR1 and various MBP-βCAs were transiently expressed in N. benthamiana by agroinfiltration and pulled-down with amylose resin. The panel shows GFP-NPR1 detected by immunoblot before treatment with resin. In the case of the empty vector, the image was cut to show that the lane developed over a shorter period of time. Ponceau-S staining of the nitrocellulose membrane is shown as a loading control. (B) The equivalent experiment, one day after treatment with 1 mM SA. (C) Expression of βCAs-MBP detected by immunoblot analysis before treatment with resin. (D) The equivalent experiment, one day after treatment with 1 mM SA. (E) Co-sedimentation of NPR1 with βCAs after purification with amylose resin; the sedimented fractions were decorated with the indicated antibodies. (F) The equivalent experiment, one day after treatment with 1 mM SA. (PDF) [file pone.0181820.s009.pdf]

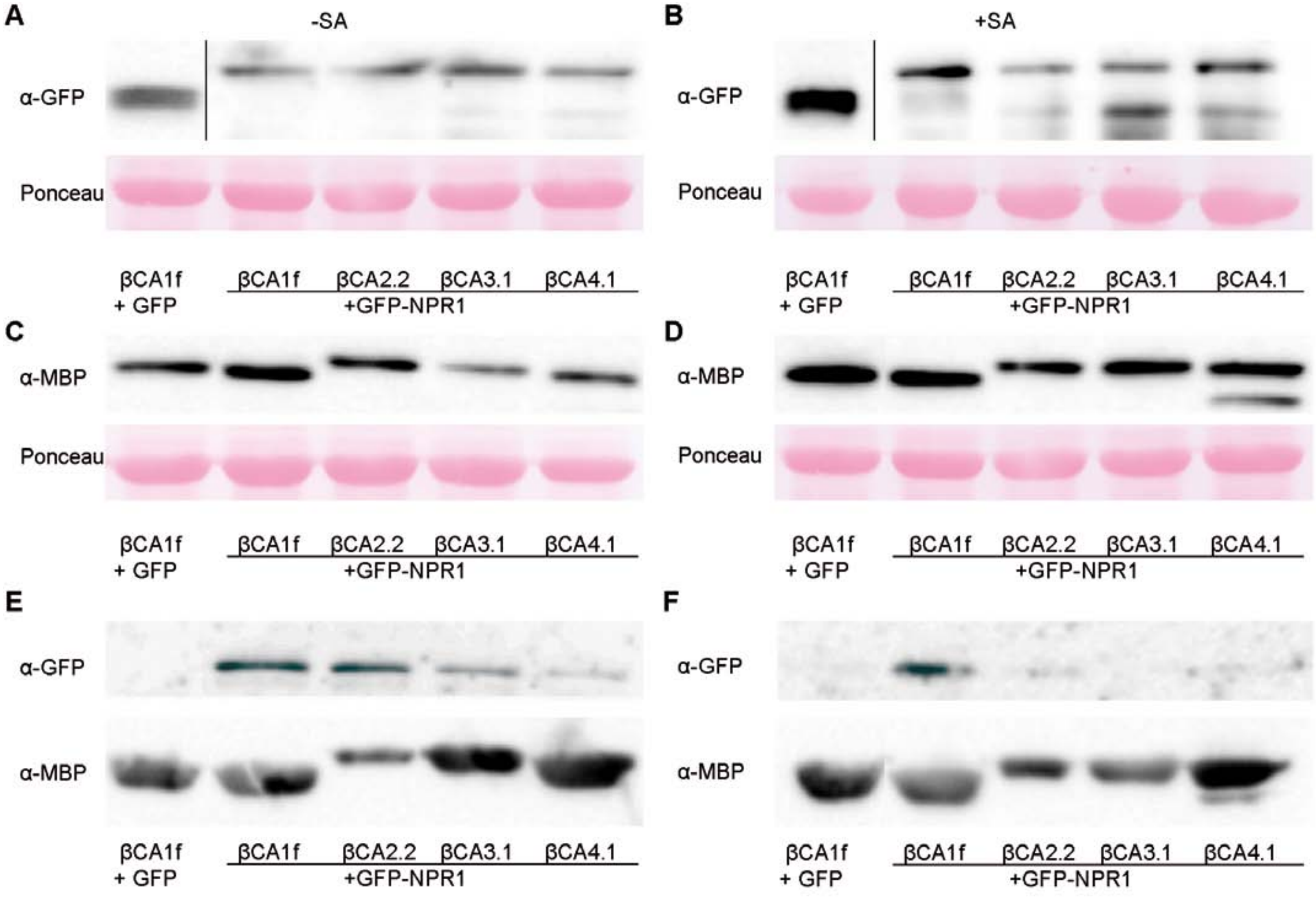

S9 Fig. Controls for co-sedimentations. (A) *NPR1-GFP* and various *βCAs-MBP* were transiently expressed in *N. benthamiana* by agroinfiltration and pulled-down with amylose resin. The panel shows NPR1-GFP detected by immunoblot before treatment with resin. In the case of the empty vector, the image was cut to show that the lane developed over a shorter period of time. Ponceau-S staining of the nitrocellulose membrane is shown as a loading control. (B) The equivalent experiment, one day after treatment with 1 mM SA. (C) Expression of βCAs-MBP detected by immunoblot analysis before treatment with resin. (D) The equivalent experiment, one day after treatment with 1 mM SA. (E) Co-sedimentation of NPR1 with βCAs after purification with amylose resin; the sedimented fractions were decorated with the indicated antibodies. (F) The equivalent experiment, one day after treatment with 1 mM SA.
