# Supplementary material for: β-carbonic anhydrases play a role in salicylic acid perception in Arabidopsis
Source: PLoS One. 2017 Jul 28;12(7):e0181820. doi: 10.1371/journal.pone.0181820 (PMC5533460; doi:10.1371/journal.pone.0181820)
Supplement: S10 Fig — Selected cDNAs from βCAs were transformed into Arabidopsis under the control of the 35S promoter and fused to GFP. (A) N-terminal fusions; the cDNAs of proteins that interact with both NPR1 and NRB4 were selected, and the response of the transgenic lines to BTH was measured in terms of weight, as in Fig 2A. (B) Response of the N-terminal fusions to SA and BTH in terms of Pto growth. Asterisks indicate statistically significant differences from the mock treatment (P < 0.05 one asterisk, P < 0.01 two) using the Student’s t test (one tail). (C) C-terminal fusions. Representative cDNAs of each gene were selected due to their chloroplastic or unknown localizations, and the response of the transgenic lines to BTH was measured. (D) Response of the C-terminal fusions to SA and BTH in terms of Pto growth. Three independent, homozygous lines were selected for each cDNA. In the case of βCA1f, no homozygous line could be recovered, and the progeny of one transgenic plant per line were used. (PDF) [file pone.0181820.s010.pdf]

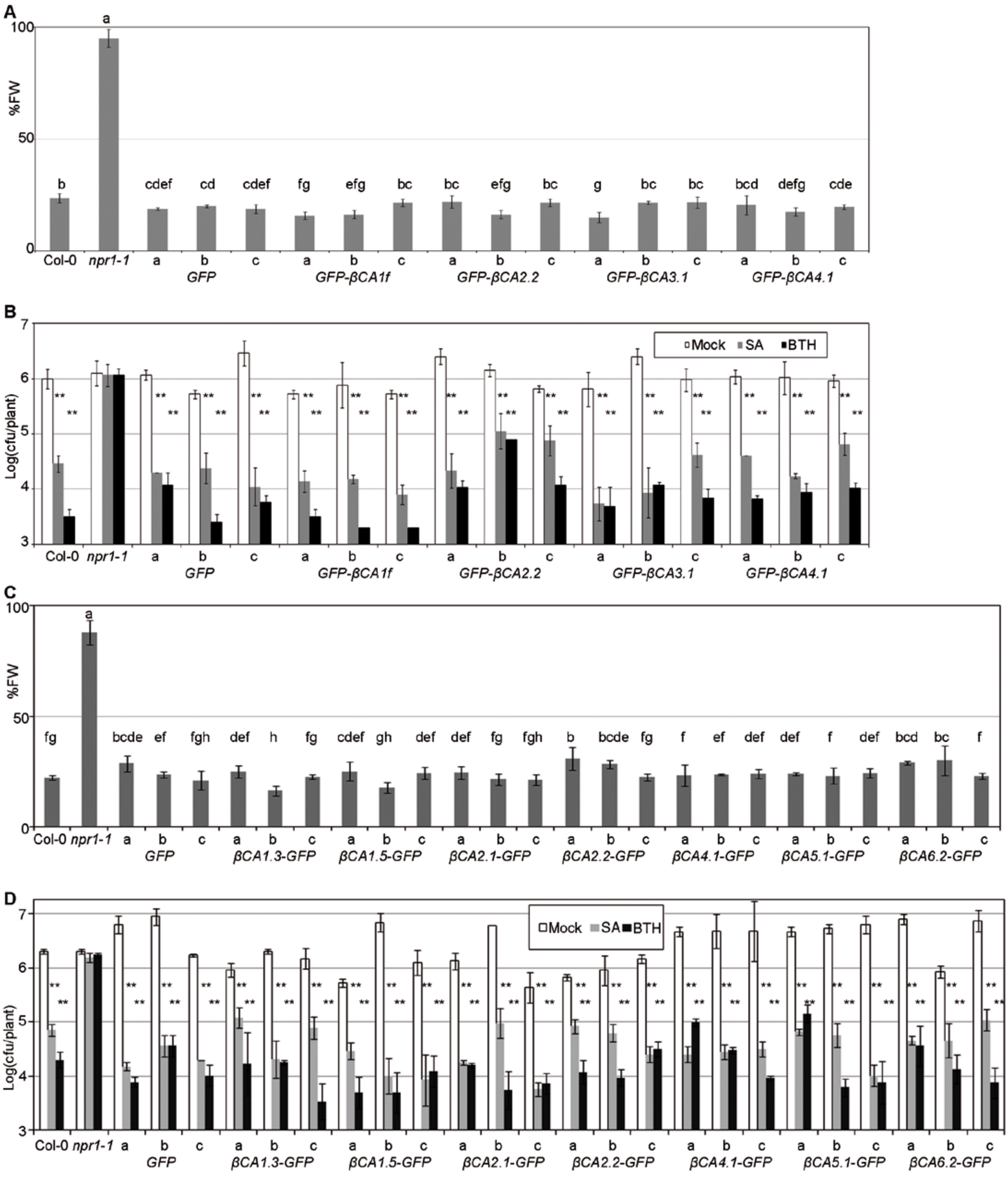

A
%FW
Col-0
npr1-1
GFP
GFP-βCA1f
GFP-βCA2.2
GFP-βCA3.1
GFP-βCA4.1
B
Log(cfu/plant)
Mock
SA
BTH
C
βCA1.3-GFP
βCA1.5-GFP
βCA2.1-GFP
βCA2.2-GFP
βCA4.1-GFP
βCA5.1-GFP
βCA6.2-GFP
D

S10 Fig. Phenotypes of the transgenic βCA lines. Selected cDNAs from βCAs were transformed into Arabidopsis under the control of the 35S promoter and fused to GFP. (A) N-terminal fusions; the cDNAs of proteins that interact with both NPR1 and NRB4 were selected, and the response of the transgenic lines to BTH was measured in terms of weight, as in Fig 2A. (B) Response of the N-terminal fusions to SA and BTH in terms of *Pto* growth. Asterisks indicate statistically significant differences from the mock treatment ($P < 0.05$ one asterisk, $P < 0.01$ two) using the Student's t test (one tail). (C) C-terminal fusions. Representative cDNAs of each gene were selected due to their chloroplastic or unknown localizations, and the response of the transgenic lines to BTH was measured. (D) Response of the C-terminal fusions to SA and BTH in terms of *Pto* growth. Three independent, homozygous lines were selected for each cDNA. In the case of βCA1f, no homozygous line could be recovered, and the progeny of one transgenic plant per line were used.
