# Supplementary material for: β-carbonic anhydrases play a role in salicylic acid perception in Arabidopsis
Source: PLoS One. 2017 Jul 28;12(7):e0181820. doi: 10.1371/journal.pone.0181820 (PMC5533460; doi:10.1371/journal.pone.0181820)
Supplement: S11 Fig — (A) Phenotypes of βca5 plants. The progeny of a heterozygous βca5-3 plant were sown individually, and the photograph was taken four weeks later. Of the 30 plants in the photograph, seven are homozygous for the T-DNA insertion in βCA5, corresponding to the smaller plants. These smaller plants were later checked using PCR markers and found to be homozygous. (B) Close-up view of a homozygous βca5-3 plant, corresponding to the plant in the bottom left corner of “A”. (C) The homozygous βca5-3 plants bolt, but the flowers do not produce fruits. (D) Close-up view of a fertile, heterozygous βca5-3 flower. (E) Close-up view of a homozygous βca5-3 flower. (PDF) [file pone.0181820.s011.pdf]

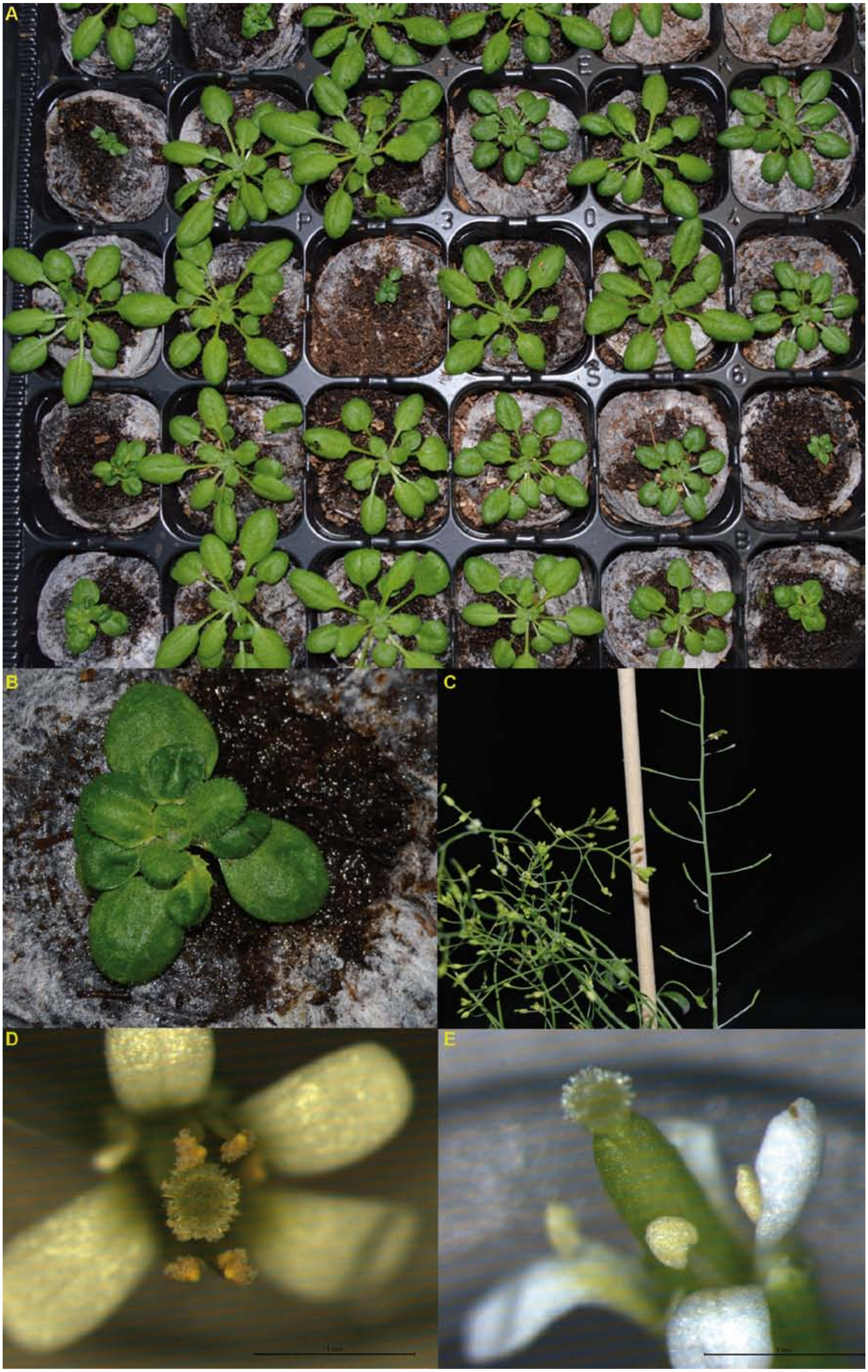
A
B
C
D
E

**S11 Fig. The *βca5* mutant is sterile.** (A) Phenotypes of *βca5* plants. The progeny of a heterozygous *βca5-3* plant were sown individually, and the photograph was taken four weeks later. Of the 30 plants in the photograph, seven are homozygous for the T-DNA insertion in βCA5, corresponding to the smaller plants. These smaller plants were later checked using PCR markers and found to be homozygous. (B) Close-up view of a homozygous *βca5-3* plant, corresponding to the plant in the bottom left corner of "A". (C) The homozygous *βca5-3* plants bolt, but the flowers do not produce fruits. (D) Close-up view of a fertile, heterozygous *βca5-3* flower. (E) Close-up view of a homozygous *βca5-3* flower.
