# Supplementary material for: β-carbonic anhydrases play a role in salicylic acid perception in Arabidopsis
Source: PLoS One. 2017 Jul 28;12(7):e0181820. doi: 10.1371/journal.pone.0181820 (PMC5533460; doi:10.1371/journal.pone.0181820)
Supplement: S12 Fig — (A) The response of the βCAs null alleles and their combinations to BTH was measured in terms of weight, as in Fig 2A. (B) Response of the null alleles to SA and BTH in terms of Pto growth. Fig 6B shows only the most important genotypes of this experiment. (C) Response to Pto(avrRpm1). (D) Response to Pto(avrRpt2). (E) Response to Pto(avrPphB). (F) Response to Pto(avrRps4). (G) Response to Pto(hopZ1a). Fig 6C shows condensed information from panels “C” to “G”. (PDF) [file pone.0181820.s012.pdf]

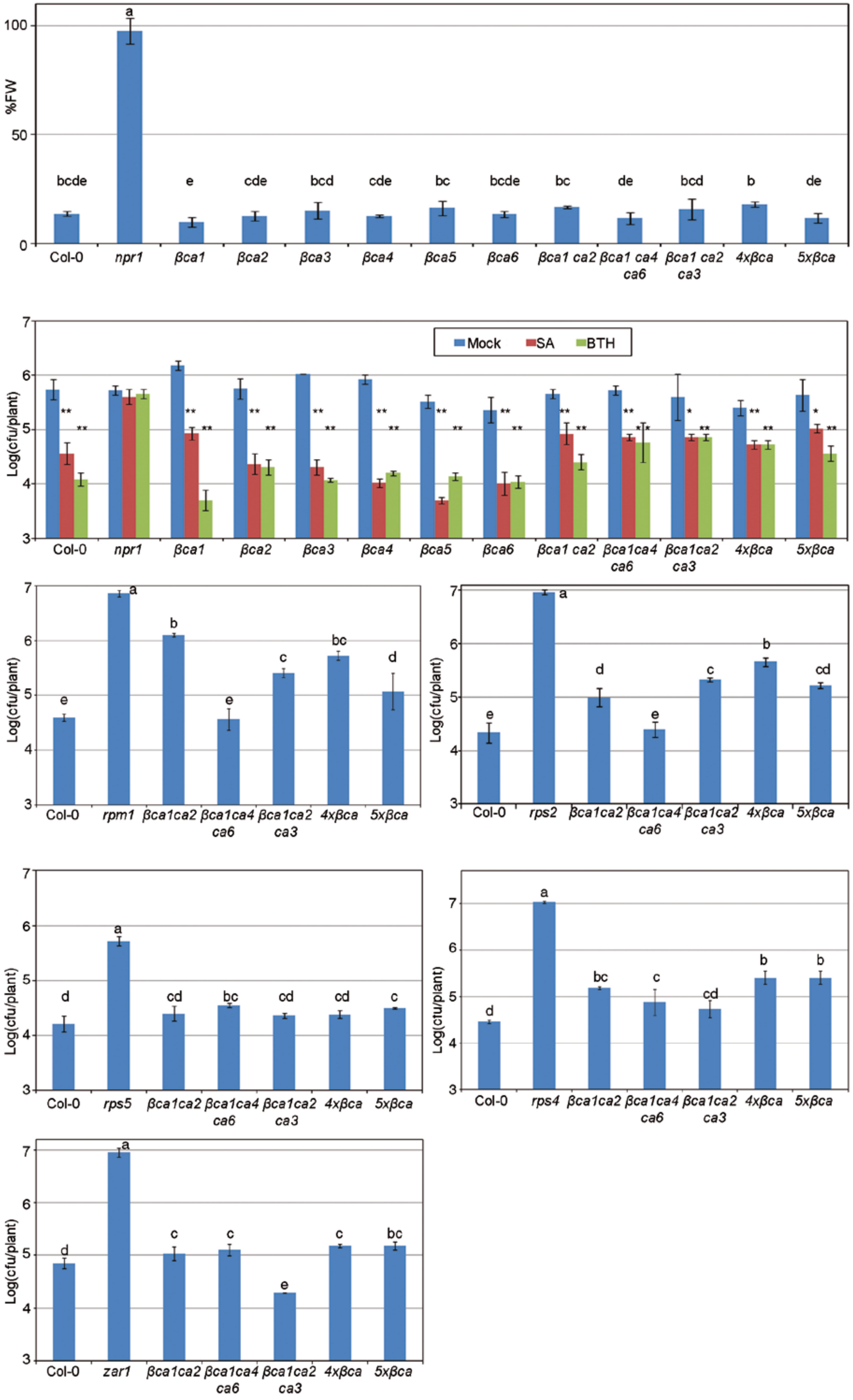
%FW
100
50
0
a
bcde
e
cde
bcd
cde
bc
bcde
bc
de
bcd
b
de
Col-0
npr1
βca1
βca2
βca3
βca4
βca5
βca6
βca1 ca2
βca1 ca4 ca6
βca1 ca2 ca3
4xβca
5xβca
Mock
SA
BTH
Log(cfu/plant)
rpm1
rps2
rps5
rps4
zar1
βca1ca2
βca1ca4 ca6
βca1ca2 ca3

S12 Fig. Additional phenotypes of the T-DNA insertion lines (I). (A) The response of the βCAs null alleles and their combinations to BTH was measured in terms of weight, as in Fig 2A. (B) Response of the null alleles to SA and BTH in terms of *Pto* growth. Fig 6B shows only the most important genotypes of this experiment. (C) Response to *Pto(avrRpm1)*. (D) Response to *Pto(avrRpt2)*. (E) Response to *Pto(avrPphB)*. (F) Response to *Pto(avrRps4)*. (G) Response to *Pto(hopZ1a)*. Fig 6C shows condensed information from panels "C" to "G".
