# Supplementary material for: β-carbonic anhydrases play a role in salicylic acid perception in Arabidopsis
Source: PLoS One. 2017 Jul 28;12(7):e0181820. doi: 10.1371/journal.pone.0181820 (PMC5533460; doi:10.1371/journal.pone.0181820)
Supplement: S13 Fig — Immunoblot analysis with anti-PR1 [28], in Col-0 (A) and the quintuple βca mutant (B) after 1 mM SA treatment. Samples were taken before, and at the indicated days after the spray. (PDF) [file pone.0181820.s013.pdf]

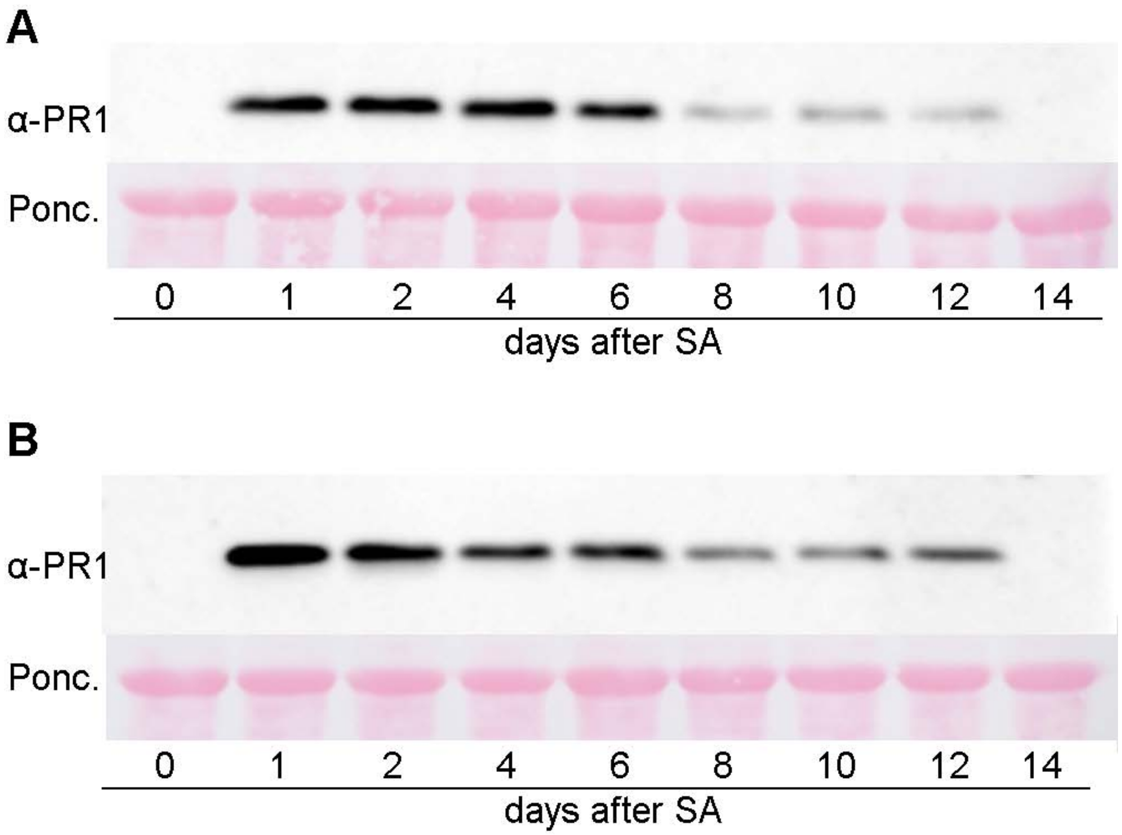


**S13 Fig. Induction of PR1 in Col-0 and in the quíntuple mutant.** Immunoblot analysis with anti-PR1 (Canet *et al.*, 2010a), in Col-0 (A) and the quintuple βca mutant (B) after 1 mM SA treatment. Samples were taken before, and at the indicated days after the spray.
