# Supplementary material for: β-carbonic anhydrases play a role in salicylic acid perception in Arabidopsis
Source: PLoS One. 2017 Jul 28;12(7):e0181820. doi: 10.1371/journal.pone.0181820 (PMC5533460; doi:10.1371/journal.pone.0181820)
Supplement: S14 Fig — (A) The single T-DNA insertion lines were grown on MS plates, and the photograph was taken at day 7 after germination. (B) The same genotypes as in “A”, grown on MS with 300 μM SA. (C) Lines with multiple T-DNA insertions grown on MS plates. (D) The same genotypes as in “C”, grown on MS with 300 μM SA. Quantification of the response of βca1 βca2 βca3 βca4 βca6 (5x βca) and its controls is shown in Fig 6E. (PDF) [file pone.0181820.s014.pdf]

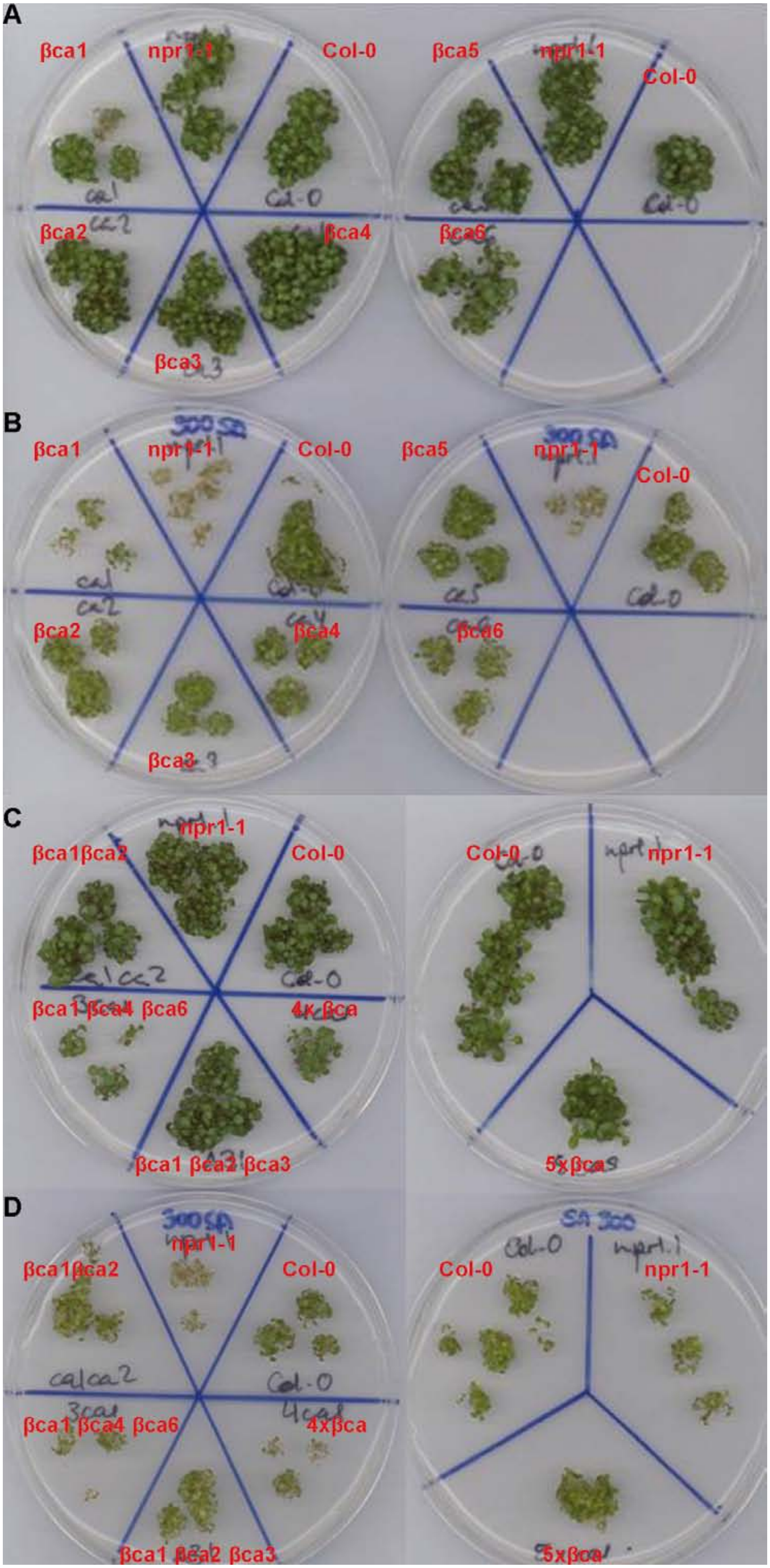
A
βca1
npr1-1
Col-0
βca2
βca4
βca3
βca5
npr1-1
Col-0
βca6
B
300 SA
βca1
npr1-1
Col-0
βca2
βca4
βca3
300 SA
βca5
npr1-1
Col-0
βca6
C
npr1-1
βca1βca2
Col-0
βca1 βca4 βca6
4x βca
βca1 βca2 βca3
Col-0
npr1-1
5xβca
D
300 SA
npr1-1
βca1βca2
Col-0
βca1 βca4 βca6
4xβca
βca1 βca2 βca3
SA 300
Col-0
npr1-1
5xβca

S14 Fig. Additional phenotypes of the T-DNA insertion lines (II). (A) The single T-DNA insertion lines were grown on MS plates, and the photograph was taken at day 7 after germination. (B) The same genotypes as in “A”, grown on MS with 300 µM SA. (C) Lines with multiple T-DNA insertions grown on MS plates. (D) The same genotypes as in “C”, grown on MS with 300 µM SA. Quantification of the response of *βca1 βca2 βca3 βca4 βca6* (*5x βca*) and its controls is shown in Fig 6E.
