# Supplementary material for: β-carbonic anhydrases play a role in salicylic acid perception in Arabidopsis
Source: PLoS One. 2017 Jul 28;12(7):e0181820. doi: 10.1371/journal.pone.0181820 (PMC5533460; doi:10.1371/journal.pone.0181820)
Supplement: S15 Fig — (A) SA binding of half of the cloned βCAs. Purified recombinant proteins were incubated with 4-AzSA, followed by UV light treatment; 4-AzSA-cross-linked proteins were detected by immunoblot analysis with antibody against SA. An immunoblot with anti-MBP is shown as a control for protein input. The first line corresponds to a negative control where UV light was omitted. (B) SA binding of the remaining cloned βCAs. (C) Behavior of GFP alone under mock and SA conditions. Note that GFP is localized to the cytosol and nucleus (not chloroplast), and there is no change upon SA treatment. (D) Changes in the localization of βCA1.3-GFP upon SA treatment. Stable transgenic Arabidopsis plants were observed under a confocal microscope one day after treatment. (E) Relative abundance of βCA1f and NPR1 when expressed from the same promoter. Stable transgenic Arabidopsis plants harboring GFP, GFP-βCA1f, and GFP-NPR1 were subject to immunoblot analysis using an anti-GFP antibody. The letters indicate independent lines. In the case of βCA1f, the progeny of a heterozygous plant were analyzed, since no homozygous plants were identified. In the case of NPR1, line “a” is in the npr1-70 background [56], and line “b” is in the npr1-1 background (this work). A NPR1-GFP line in the WT background was also tested [57]. Except for the NPR1-GFP line, the remaining constructs are in the same plasmid backbone, pMDC43. Ponceau staining is shown below as a loading control. (PDF) [file pone.0181820.s015.pdf]

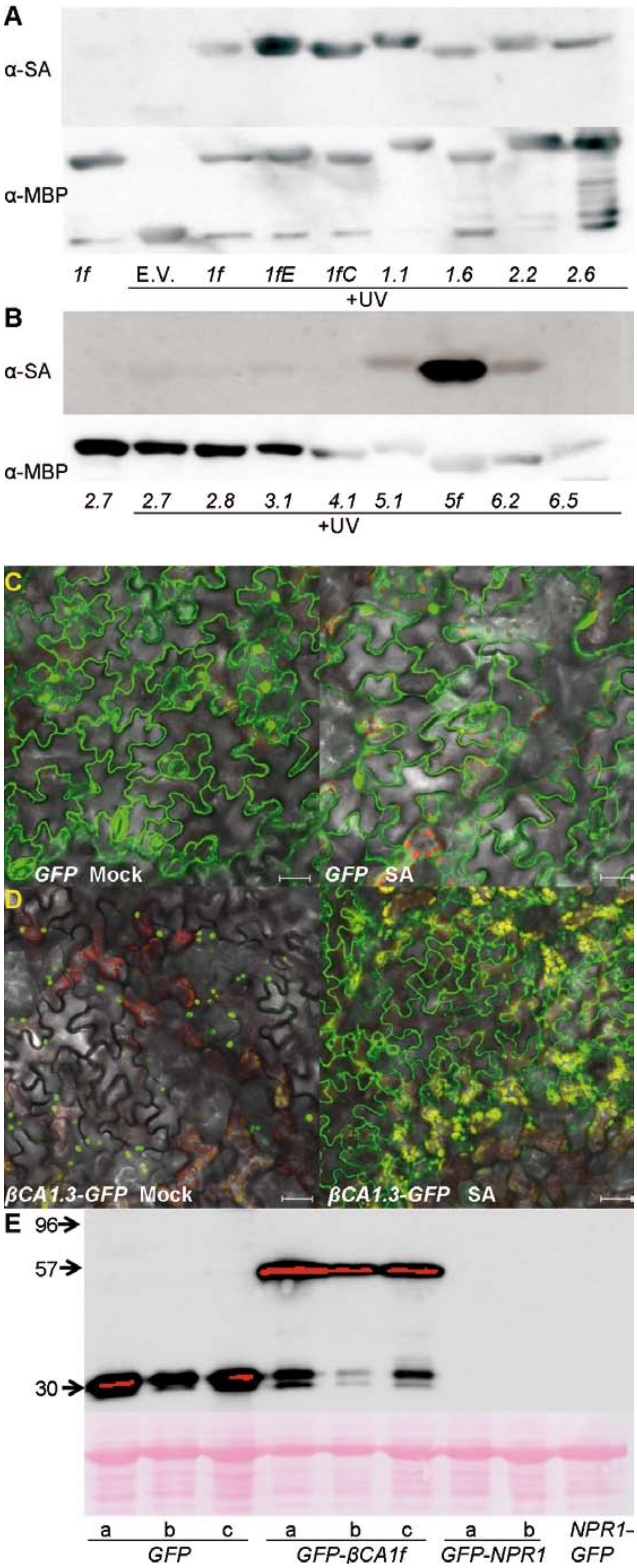

A
α-SA
α-MBP
1f
E.V.
1f
1fE
1fC
1.1
1.6
2.2
2.6
+UV
B
α-SA
α-MBP
2.7
2.7
2.8
3.1
4.1
5.1
5f
6.2
6.5
+UV
C
GFP Mock
GFP SA
D
βCA1.3-GFP Mock
βCA1.3-GFP SA
E
96
57
30
a
b
c
a
b
c
a
b
GFP
GFP-βCA1f
GFP-NPR1
NPR1-GFP

**S15 Fig. Additional controls for Fig 8.** (A) SA binding of half of the cloned βCAs**.** Purified recombinant proteins were incubated with 4-AzSA, followed by UV light treatment; 4-AzSA-cross-linked proteins were detected by immunoblot analysis with antibody against SA. An immunoblot with anti-MBP is shown as a control for protein input. The first line corresponds to a negative control where UV light was omitted. (B) SA binding of the remaining cloned βCAs. (C) Behavior of GFP alone under mock and SA conditions. Note that GFP is localized to the cytosol and nucleus (not chloroplast), and there is no change upon SA treatment. (D) Changes in the localization of βCA1.3-GFP upon SA treatment. Stable transgenic Arabidopsis plants were observed under a confocal microscope one day after treatment. (E) Relative abundance of βCA1f and NPR1 when expressed from the same promoter. Stable transgenic Arabidopsis plants harboring GFP, GFP-βCA1f, and GFP-NPR1 were subject to immunoblot analysis using an anti-GFP antibody. The letters indicate independent lines. In the case of βCA1f, the progeny of a heterozygous plant were analyzed, since no homozygous plants were identified. In the case of NPR1, line "a" is in the *npr1-70* background (Canet *et al.*, 2012a), and line "b" is in the *npr1-1* background (this work). A *NPR1-GFP* line in the WT background was also tested (Mou *et al.*, 2003). Except for the *NPR1-GFP* line, the remaining constructs are in the same plasmid backbone, pMDC43. Ponceau staining is shown below as a loading control.
