# Supplementary material for: β-carbonic anhydrases play a role in salicylic acid perception in Arabidopsis
Source: PLoS One. 2017 Jul 28;12(7):e0181820. doi: 10.1371/journal.pone.0181820 (PMC5533460; doi:10.1371/journal.pone.0181820)
Supplement: S16 Fig — Esterase activity of the cloned βCAs. Esterase activity was determined as described [64]. Serial dilutions of commercial esterase (Ref 75742 SIGMA) were included as an internal control. (PDF) [file pone.0181820.s016.pdf]

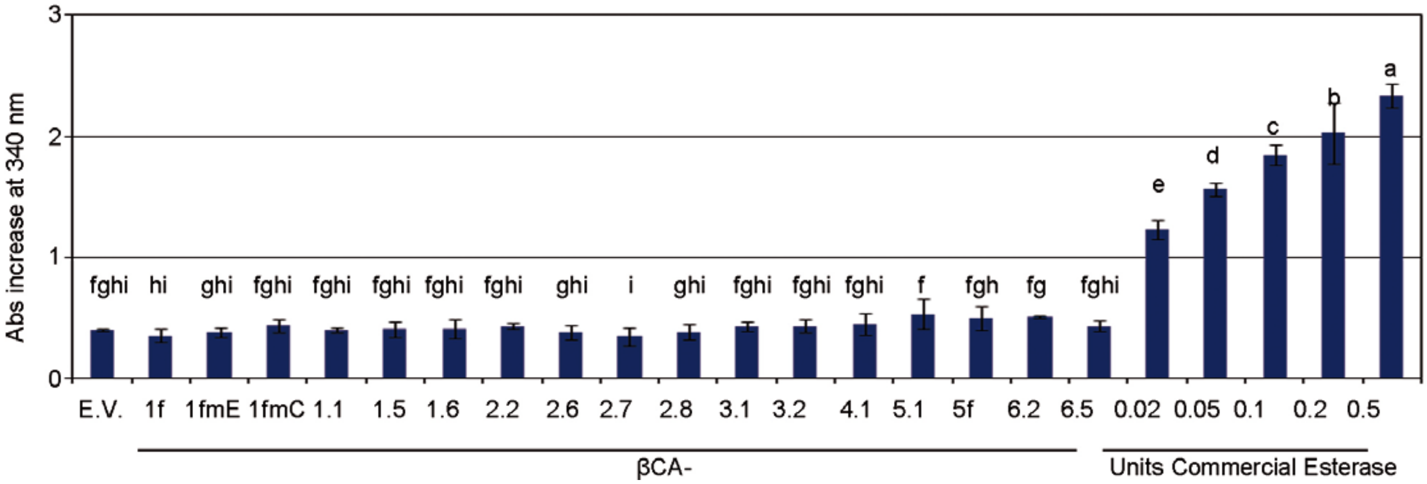

S16 Fig. Esterase activity of the βCAs. Esterase activity of the cloned βCAs. Esterase activity was determined as described (Kaul *et al.*, 2011). Serial dilutions of commercial esterase (Ref 75742 SIGMA) were included as an internal control.
