# Supplementary material for: β-carbonic anhydrases play a role in salicylic acid perception in Arabidopsis
Source: PLoS One. 2017 Jul 28;12(7):e0181820. doi: 10.1371/journal.pone.0181820 (PMC5533460; doi:10.1371/journal.pone.0181820)
Supplement: S1 Table — (PDF) [file pone.0181820.s017.pdf]

**S1 Table. Primers used in this work.**

| Number | Sequence | Objective |
|---|---|---|
| TP547 | GGGGACAAGTTTGTACAAAAAAGCAGGCTTCACCATGTCG ACCGCTCCTCTCTCCGGCTTC | Cloning CA1.3F |
| TP548 | GGGGACCACTTTGTACAAGAAAGCTGGGTGTACAGAGCTA GTTTCGGAGAGGCCAAA | Cloning CA1.3R |
| TP549 | GGGGACAAGTTTGTACAAAAAAGCAGGCTTCACCATGGTC CCCTTTTGGACTACAGTTTC | Cloning CA2.1F |
| TP550 | GGGGACAAGTTTGTACAAAAAAGCAGGCTTCACCATGGGA AACGAATCATATGAAGAC | Cloning CA2.2F |
| TP551 | GGGGACCACTTTGTACAAGAAAGCTGGGTGTATAGAATGA ACGGGGGAAATTC | Cloning CA2.1R |
| TP552 | GGGGACAAGTTTGTACAAAAAAGCAGGCTTCACCATGGGA ACCGAAGCATACGACGAG | Cloning CA1.5F |
| TP553 | TCCATCCTGGAGATGCCTTC | ca2 T-DNA marker |
| TP554 | CATCGGCCACATTGGTCTTC | ca2 T-DNA marker |
| TP578 | TAGCCGGTGTGGAGGAATTCAAGC | ca5 T-DNA marker |
| TP579 | CAGCGATTCCACTGCCTTCCTT | ca5 T-DNA marker |
| TP624 | TTGGATCGGATCGGTTTCAGC | ca6 T-DNA Marker |
| TP625 | TTGGTCCATTCTATCAAAAACAGGAAAA | ca6 T-DNA Marker |
| TP646 | GGGGACCACTTTGTACAAGAAAGCTGGGTTTCATACAGAG CTAGTTTCGGAG | Cloning CA1.3Rev |
| TP647 | GGGGACCACTTTGTACAAGAAAGCTGGGTTTCATCGATCT AACATGTGTTTG | Cloning CA1.6Rev |
| TP648 | GGGGACAAGTTTGTACAAAAAAGCAGGCTTCATGGGAAAC | Cloning CA2.2 for |

| | | |
|---|---|---|
| | GAATCATATGAAGAC | |
| TP649 | GGGGACCACTTTGTACAAGAAAGCTGGGTTTCATATAGAATGAACGGGGGA | Cloning CA2.2 rev |
| TP650 | ATGCGAAAGGGTACTGAATGTGTCACTAGCAAAC | Cloning CA2.7For |
| TP651 | GCTAGTGACACATTCAGTACCCTTTCGCATCGGCCA | Cloning CA2.7Rev |
| TP652 | GGCCGATGCGAAAGGGCAGTGAATGTGTCACTAGCAAAC | Cloning CA2.8For |
| TP653 | TAGTGACACATTCACTGCCCTTTCGCATCGGCCACATTG | Cloning CA2.8Rev |
| TP654 | GGGGACAAGTTTGTACAAAAAAGCAGGCTTCATGTCGACAGAGTCGTACGAAG | Cloning CA3 For |
| TP655 | GGGGACCACTTTGTACAAGAAAGCTGGGTTTTAAGACAAGGCAAAGGCAGGGGT | Cloning CA3 Rev |
| TP656 | GGGGACAAGTTTGTACAAAAAAGCAGGCTTCATGGCAACGGAATCGTACGAAGC | Cloning CA4 For |
| TP657 | GGGGACCACTTTGTACAAGAAAGCTGGGTTTTAAGAGAAGGCAAAAGCAGGAGTG | Cloning CA4 Rev |
| TP658 | GGGGACAAGTTTGTACAAAAAAGCAGGCTTCATGGCAGCCACTCCCACACACTTCTC | Cloning CA5 For |
| TP659 | GGGGACCACTTTGTACAAGAAAGCTGGGTTTCAAGACCAAACTGACCGGTCTTTAAC | Cloning CA5 Rev |
| TP660 | GGGGACAAGTTTGTACAAAAAAGCAGGCTTCATGGTTCAAGAATTAGGAATCAG | Cloning CA6 For |
| TP661 | GGGGACCACTTTGTACAAGAAAGCTGGGTTTCAACTCCATATCTCTCTGTCTCTCT | Cloning CA6.1 Rev |
| TP662 | GGGGACCACTTTGTACAAGAAAGCTGGGTTTCAACTCCATATCTCTCTGTCTGAAA | Cloning CA6.3 Rev |

| | | |
|---|---|---|
| TP663 | GGGGACCACTTTGTACAAGAAAGCTGGGTTTCATAGTAGC CTCCCTTCAAAG | Cloning CA2.5 REV |
| TP670 | ATACATCAGTGTTGTTTTGTCTATCAATTGCA | ca5.3 T-DNA marker |
| TP671 | CCACGTCTCTCCTTAATCTCCAAACTCA | ca5.3 T-DNA marker |
| TP678 | GGGGACAAGTTTGTACAAAAAAGCAGGCTTCTGAGTAGCT GAAACCCA | Stops for N terminal fusion |
| TP679 | GGGGACCACTTTGTACAAGAAAGCTGGGTTTCAGCTACTC AGAAGCC | Stops for N terminal fusion |
| TP680 | GGGGACAAGTTTGTACAAAAAAGCAGGCTTCGAAGGAGAT AGAACCATG | ATGs for C terminal fusion |
| TP681 | GGGGACCACTTTGTACAAGAAAGCTGGGTGCATGGTTCTA TCTCCTTC | ATGs for C terminal fusion |
| TP684 | GGGGACAAGTTTGTACAAAAAAGCAGGCTTCATGGCTTCA GGAAAGACACCTGG | For CA5s |
| TP689 | GGGGACAAGTTTGTACAAAAAAGCAGGCTTCGAAGGAGAT AGAACCATGGCTCCTGCATTCGGAAAATGTTTC | Ct fusions of CA4 |
| TP690 | GGGGACCACTTTGTACAAGAAAGCTGGGTGAGAGAAGGCA AAAGCAGGAGTGGTC | Ct fusions of CA4 |
| TP691 | GGGGACAAGTTTGTACAAAAAAGCAGGCTTCGAAGGAGAT AGAACCATGGCAGCCACTCCCACACACTTCTC | Ct fusions of CA5 |
| TP692 | GGGGACCACTTTGTACAAGAAAGCTGGGTGAGACCAAACT GACCGGTCTTTAACAGC | Ct fusions of CA5 |
| TP693 | GGGGACAAGTTTGTACAAAAAAGCAGGCTTCGAAGGAGAT AGAACCATGGCGTTTACACTAGGTGGAAGAGC | Ct fusions of CA6 |
| TP694 | GGGGACCACTTTGTACAAGAAAGCTGGGTGACTCCATATC | Ct fusions of CA6 |

| | | |
|---|---|---|
| | TCTCTGTCTGAAATATAG | |
| TP701 | GGCGGAGCTAAAGGAGCTTGACTCAAGCAATTC | ca4 TDNA marker |
| TP702 | ACCAGGTTGGAAATTCAAGATGTGAGATGGACAAA | ca4 TDNA marker |
| TP784 | GGCGTTGGAGCAGCCATTGCA | Introduce a point mutation E204A in CA1 |
| TP785 | AAGGTGTAAGACCGCGTATGC | Introduce a point mutation E204A in CA1 |
| TP787 | AAGCCCTTTGATCCCACCAGA | Introduce a point mutation C223S in CA1 |
| TP788 | GGGGACAAGTTTGTACAAAAAAGCAGGCTTCACGGTTGCAGCGGCAAAGGTGG | Amplify CA1f for point mutation |
| TP810 | GTGGTGATAGGACACAGTGCATCTGGTGGG | Introduce a point mutation C223S in CA1 |
| TP814 | TGCCAAGAGCCAGACCCCCAAGGTTTTGA | ca3 TDNA marker |
| TP815 | TCCTGCTTGATCCTGTTCTTGGCCGGTGC | ca3 TDNA marker |
